# Role of a LORELEI- like gene from *Phaseolus vulgaris* during a mutualistic interaction with *Rhizobium tropici*

**DOI:** 10.1371/journal.pone.0294334

**Published:** 2023-12-07

**Authors:** Edgar Pascual-Morales, Pamela Jiménez-Chávez, Juan E. Olivares-Grajales, Luis Sarmiento-López, Wylly R. García-Niño, Aline López-López, Paul H. Goodwin, Janet Palacios-Martínez, Ana I. Chávez-Martínez, Luis Cárdenas

**Affiliations:** 1 Departamento de Biología Molecular de Plantas, Instituto de Biotecnología, Universidad Nacional Autónoma de México, Cuernavaca, Morelos, México; 2 Departamento de Biociencias y Agrotecnología, Centro de Investigación en Química Aplicada, Saltillo, Coahuila, México; 3 School of Environmental Sciences, University of Guelph, Guelph, Ontario, Canada; Agri Biotech Foundation and Retired Professor, University of Hyderabad, INDIA

## Abstract

Reactive oxygen species (ROS), produced by NADPH oxidases known as RBOHs in plants, play a key role in plant development, biotic and abiotic stress responses, hormone signaling, and reproduction. Among the subfamily of receptor-like kinases referred to as CrRLK, there is FERONIA (FER), a regulator of RBOHs, and FER requires a GPI-modified membrane protein produced by LORELEI (LRE) or LORELEI-like proteins (LLG) to reach the plasma membrane and generate ROS. In Arabidopsis, *AtLLG1* is involved in interactions with microbes as AtLLG1 interacts with the flagellin receptor (FLS2) to trigger the innate immune response, but the role of LLGs in mutualistic interactions has not been examined. In this study, two *Phaseolus vulgaris* LLG genes were identified, *PvLLG2* that was expressed in floral tissue and *PvLLG1* that was expressed in vegetative tissue. Transcripts of *PvLLG1* increased during rhizobial nodule formation peaking during the early period of well-developed nodules. Also, *P*. *vulgaris* roots expressing *pPvLLG1*:*GFP-GUS* showed that this promoter was highly active during rhizobium infections, and very similar to the subcellular localization using a construct *pLLG1*::*PvLLG1-Neon*. Compared to control plants, *PvLLG1* silenced plants had less superoxide (O^2-^) at the root tip and elongation zone, spotty hydrogen peroxide (H_2_O_2_) in the elongation root zone, and significantly reduced root hair length, nodule number and nitrogen fixation. Unlike control plants, *PvLLG1* overexpressing plants showed superoxide beyond the nodule meristem, and significantly increased nodule number and nodule diameter. *PvLLG1* appears to play a key role during this mutualistic interaction, possibly due to the regulation of the production and distribution of ROS in roots.

## Introduction

An important group of plant root mutualistic microbes are the nitrogen-fixing bacteria in the *Rhizobiaceae* family, which convert the diatomic nitrogen (N_2_) to ammonium ion (NH_4_)^+^ inside root nodules of leguminous plants [1]. During this interaction, the plant releases phenolic metabolites, such as flavonoids into the rhizosphere, which are specifically recognized by rhizobia. This induce the synthesis and secretion of Nod factors by rhizobia which are recognized by specific plant membrane receptors initiating a signal cascade inducing responses, such as Ca^2+^ fluxes, membrane depolarization, ROS production, cytoskeleton rearrangement and altered gene expression [2, 3]. During nodulation, infection starts with a swelling of the root hair tip, followed by root hair curling and infection thread (IT) formation to allow the bacteria to gain access to root cortex cells where the nodule primordium develops into the nitrogen fixing nodule, all of which requires root morphological changes [4].

In plants, ROS are produced by NADPH oxidases, known as RboHs (Respiratory Burst Oxidase Homologue). In *Arabidopsis*, RboHs have many important roles, such as in plant cell development and interactions with microorganisms. For instance, deletion mutants of *AtRbohC* have impaired root hairs that burst immediately after emergence [5], and deletion mutants of *AtRbohD* and *AtRohF* are more susceptible to infection by the pathogens *Pseudomonas syringae* pv. *tomato* and *Peronospora parasitica* [6]. RBOHs can be regulated by Ca^2+^, phosphorylation, and small RAC/ROP GTPases [7]. RBOHs are also regulated via FER-RAC-ROP pathway by a subfamily of receptor-like kinases (CrRLKs), made up of 17 members with the first one identified in *Catharanthus roseus* [8–10]. These receptors have two extracellular malectin domains involved in carbohydrate binding, such as cell wall sugars to sense cell wall integrity [11, 12]. Other CrRLKs are related to growth of root hairs and pollen tubes [13]. This might have some relationship to mutualistic root interactions as mutations resulting in the overexpression of the CrRLK gene, *ANX1- OX*, that caused invaginated apical pollen tubes, which appeared similar to initial rhizobial infection threads in root hairs [14–16]. Another widely studied CrRLK is FERONIA (FER), whose expression increases in regions of greater cell elongation, such as root hairs [13]. Both FER and NADPH oxidase deletion mutants in Arabidopsis had a similar phenotype with root hairs that burst immediately after emerging, which is likely because they are linked since FER activates GEF which allows the activation of RAC/ROP GTPases that recruits and activate RboH to produce ROS [5, 17–19]. Since FER is constitutively expressed in most plant tissues, it has been proposed that its binding with different RALFs (rapid alkalinization factors) regulate its activity to determine its roles in different plant tissues, such as the FER-RALF1 interaction regulating cell expansion and the FER-RALF23 interaction regulating pathogen defenses [17, 20, 21].

LORELEI (LRE) or LORELEI-like (LLG) proteins act as chaperones and coreceptors for FER [17]. In the Arabidopsis genome, there is one LRE gene (*AtLRE1*), and three *LLG* genes (*AtLLG1*, *AtLLG2*, and *AtLLG3)* [22–24]. *AtLRE1* is expressed only in the synergid, egg, and central cells of the female gametophyte cells [25, 26]. The three *LLG* genes are expressed in many tissues, but *AtLLG1* is most highly expressed in vegetative tissues, and *AtLLG2* and *AtLLG3* are most highly expressed in male reproductive tissues [17, 25, 27]. Certain LLGs can bind with RALF peptides, which can be higher affinity than with FER [28]. Combinations of differential expression and binding of LREs and LLGs with FER and RALF ligands have been proposed to determine the wide variety of roles observed for LREs and LLGs [29, 30]. LRE and FER are well known for their joint function in pollen tube reception at the interface of the synergid cell and pollen tube [31]. Another example is the regulation of the immune response to pathogens by affecting the subcellular location of PAMP receptors (Shen et al., 2017), and regulation of root hair tip growth maintaining apical ROS and Ca^2+^ gradients (Duan et al., 2010; Foreman et al., 2003; Li et al., 2015).

Since plant pathogenic and mutualistic interactions are both ROS-regulated processes, they could both involve LLG proteins acting as mediators [32–35]. LLG proteins could be directly important for ROS-regulated processes as LLG binds with FER, FER activates RboH via RAC/ROP GTPase resulting in ROS production [18, 19]. This work constitutes a first examination of the role of *LLGs* in roots during the important mutualistic interaction with *Rhizobium tropici*.

## Results

### Features of LLG genes in legumes

Using *AtLLG1*, *AtLLRE*, *AtLLG2*, and *AtLLG3* from Arabidopsis as queries against the Phytozome v.13.0 database, two genes, Phvul.005G003700 and Phvul.011G114300 were identified from *P*. *vulgaris* S1 Table. Based on their highest matches, Phvul.005G003700 was designated *PvLLG1*, and Phvul.011G114300 was designated *PvLLG2*. The same analysis was done for seven other legumes, *M*. *truncatula*, *L*. *japonicus*, *C*. *arietinum*, *G*. *max*, *L*. *culinaris and A*. *hypogea*, and *LLG1* and *LLG2* genes were identified in each legume. No genes had higher identity to *AtLLRE* than *AtLLG1*, and none had higher identity with *AtLLG3* than *AtLLG2*. Thus it appears that legumes only have homologs of *AtLLG1* and *AtLLG2*. For the legumes, each species examined had one *LLG1* and one *LLG2* gene, except for *G*. *max*, which had three *LLG1* variants and two *LLG2* variants, *A*. *hypogea* which had four *LLG2* variants, and *M*. *truncatula* which had two *LLG2* variants.

**Fig 1:**
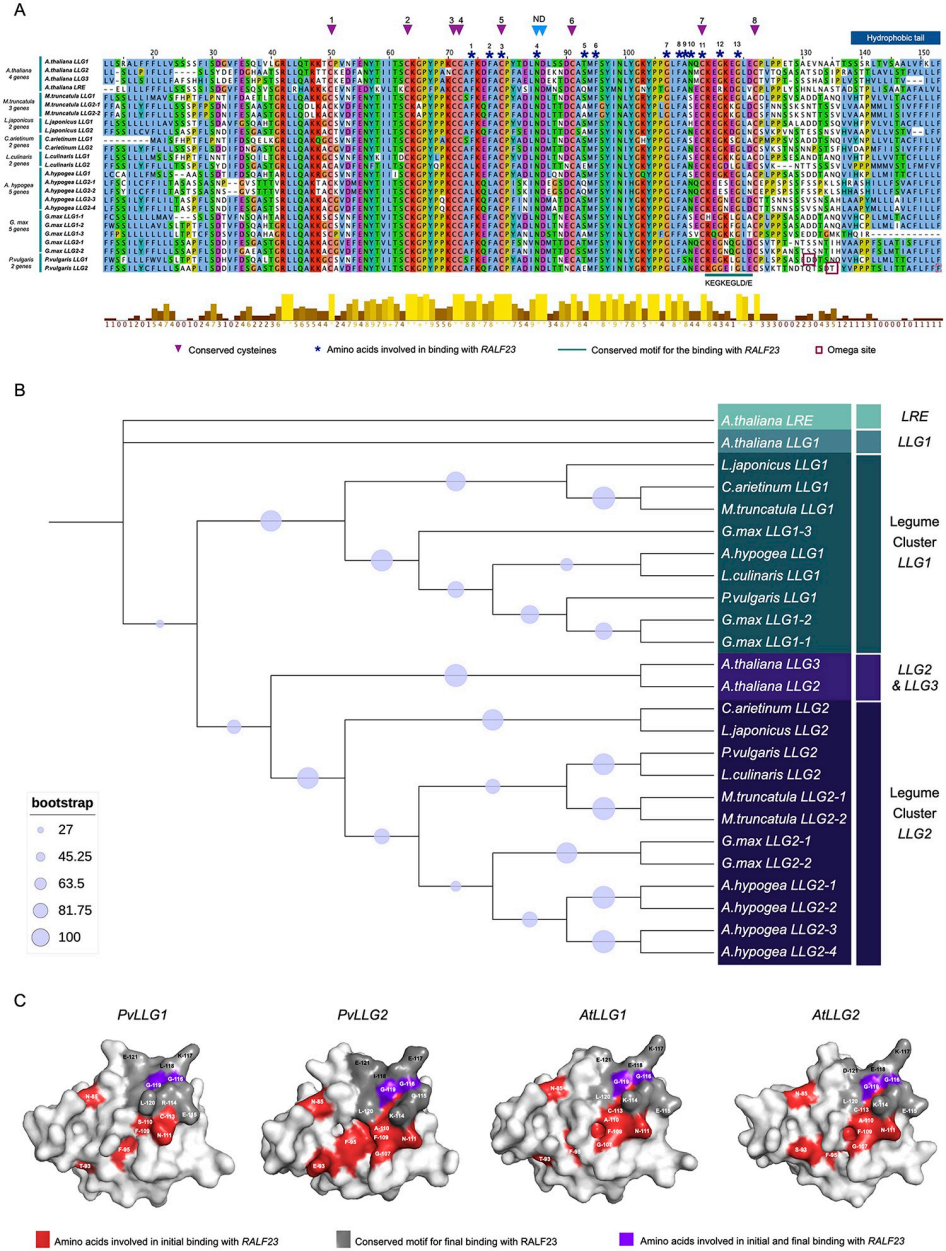
Phylogenetic analysis of LRE/LLG in the Fabaceae family and modeling of the LLG1 and LLG2 proteins. (**A**) Alignment of the amino acid sequence of LRE/LLG proteins from 7 members of Fabaceae family. The 8 conserved cysteines are indicated with purple triangles, the conserved Asn-Asp dipeptide (ND) is indicated with blue tringles, the 13 amino acids involved in binding with RALF23 are indicated with navy blue asterisks, the conserved motif KEGKEGLE/D for binding with RALF23 is indicated with a green line, the omega site for PvLLG1 and PVLLG2 is indicated with a dark red square, and the hydrophobic tail is indicated with the blue box. (**B**) Maximum Likelihood tree constructed using IQTree and iTOL software with nodes showing percent of 1000 boot strap replicates. (**C**) Surface models for LLG1 and LLG2 from *P*. *vulgaris* and *A*. *thaliana* with the exposed amino acids involved in RALF23 binding indicated in red, the exposed amino acids involved in complementary binding with RALF23 indicated in gray, and the exposed amino acids in common between the two binding motifs binding with RALF23 in purple.

The 25 predicted LLG1 and LLG2 protein sequences from legumes and the four sequences from Arabidopsis all had highly conserved 8 cysteine residues for the formation of 4 disulfide bonds (Fig 1A). For the 13 amino acids for binding of RALF23, all are completely conserved, except for sites 5, 9, 10 and 12, which had 5, 2, 3 and 3 possible amino acids, respectively. While not universal, most differences were observed between the variants of the LLG1 and LLG2 predicted proteins of *G*. *max*, *A*. *hypogea* and *M*. *truncatula*. None of the legume sequences had the arginine at number 12 of the binding amino acids motif, which is characteristic of AtLRE. The motif ‘KEGKEGLE/D’ for binding to RALF23 was conserved for all the predicted LLG proteins of Arabidopsis, but not among the predicted LLG1 and LLG2 proteins from the legumes. The only sequences that exactly matched it was CaLLG1. The amino acid sequences most different from ‘KEGKEGLE/D’ were GmLLG1-3 with 5 differences, GmLLG1-1 and GmLLG1-2 with 4 differences, and AhLLG2-1 and AhLLG2-2 with 2 differences. All of the sequences had a hydrophobic tail, except for GmLLG1-3. In addition to the previously described conserved amino acids, there were also a number of other highly conserved amino acids, R-42, L-43, K-64, P-66, A-78, M- 94, Y- 97, N-99, G-102, Y-104 and P-105. These may be conserved because of an importance in the secondary and tertiary structures of these LLG/LRE genes. The most probable omega site for GPI modification in PvLGG1 and PvLLG2 were at amino acids 131 and 135, respectively.

A tree of the predicted LLG/LRE sequences showed that all the legume sequences could be divided into two clusters, named legume LLG1 and legume LLG2 Fig 1B. The LLG/LRE predicted proteins from Arabidopsis were distinct from those of legumes. For the LLG1 cluster, there were two main subclusters. One had *L*. *japonicus*, *C*. *arietinum* and *M*. *truncatula* sequences, and the other had *G*. *max*, *A*. *hypogea*, *L*. *culinaris* and *P*. *vulgaris* sequences. The three *G*. *max* sequences were divided into LLG1-1 and LLG1-2, which were very similar, perhaps indicating a relatively recent gene duplication event, and a more distantly related LLG1-3. For the LLG2 cluster, there were two main subclusters. One had *C*. *arietinum* and *L*. *japonicus* sequences, and the other had *P*. *vulgaris*, *L*. *culinaris*, *M*. *truncatula*, *G*. *max* and *A*. *hypogea* sequences. The multiple *M*. *truncatula*, *G*. *max* and *A*. *hypogea* LLG2 sequences all clustered by species perhaps indicating relatively recent gene duplication events.

For *P*. *vulgaris*, the predicted surface models of PvLLG1 and PvLLG2 revealed clear differences in the two areas that could interact with RALF23 (Fig 1C). The surface area of PvLLG1 for the 13 aa for binding was discontinuous, notably between T-93 and N-111, whereas it formed more of a continuous surface on PvLLG2 between E-93 and N-111. The 8 aa region for RALF23 binding matching the Arabidopsis conserved KEGKEGLE/D motif was REGKLGLE in PvLLG1 and KGGEIGLE in PvLLG2 with all of those aa visible on the predicted surface models of the two proteins. The shape of the surfaces for RALF23 binding was similar in the two predicted proteins, except that there was groove in PVLLG1 between G-119 and L-120, whereas there was a groove between L-120 and K-114 in PvLLG2 with two aa for RALF23 binding (G119 and G116), within the groove. For Arabidopsis, both AtLLG1 and AtLLG2 has a similar discontinuous distribution in the surface distribution of the 13 aa for RALF23 binding S1 Fig. They both also showed a groove between L-120 and K-114 with C-113 as well as G-119 for binding being found within the groove. However, there were differences, such as T-93 and F-95 being separated in AtLLG1 while S-93 and F-95 were more continuous in AtLLG2. Overall, AtLLG1 and AtLLG2 had more similar predicted surface models related to RALF23 binding than PvLLG1 and PvLLG2, particularly for the second binding motif which would be expected with 1 versus 4 aa differences in the 8 aa motifs for complementary RALF23 binding.

### Expression of *PvLLG1* and *PvLLG2*

**Fig 2:**
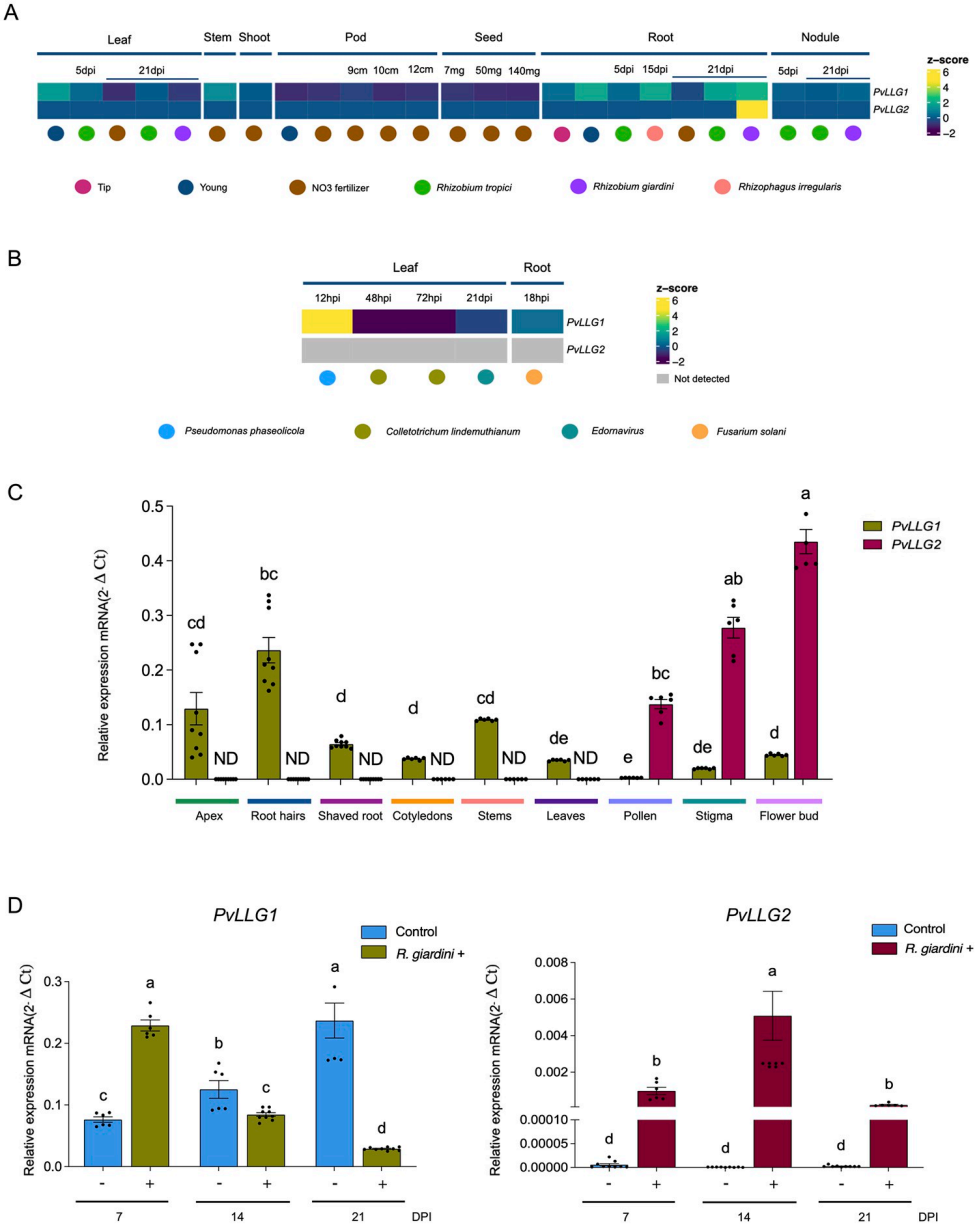
*PvLLG1* and *PvLLG2* expression in *P*. *vulgaris*. (**A**) Heatmap of expression based on data at the Phytozome v.13.0. (**B**) Heatmap of expression reported by O’Rourke *et al*. (2014) and Nanjareddy *et al*. (2017). (**C**) mRNA levels in *P*. *vulgaris* tissues determined by RT-qPCR. (D) *PvLLG1* and *PvLLG2* transcript accumulation under *R*. *giardini* inoculation. Quantification by RT-qPCR is expressed as relative expression (2^−ΔCt^) calculated with normalization to the *P*. *vulgaris* housekeeping gene, *PvEf1α*. For each sample, three biological replicates were analyzed with three technical replicates each. Different letters indicate significant differences among samples according to the Two -Way ANOVA analysis at *p* <0.0001.

The transcriptional profiles of *PvLLG1* and *PvLLG2* were used to construct a heatmap based on the transcriptional database reported by several authors [36–41] Fig 2A and 2B. Also included were the accumulation values of *PvLLG1* and *PvLLG2* transcripts during nodule development from this study. For *PvLLG1*, high Z-scores (≥ 2) were observed for 5 dpi leaves, *P*. *phaseolicola* in leaves, NO_3_ in stems, 5 dpi roots, *R*. *irregularis* in roots, *R*. *tropici* in roots, and *R*. *giardini* in roots, while low Z-scores (≥ -2) were observed for NO3 in leaves, *R*. *giardini* in leaves, 9–12 cm pods, 7–140 mg seeds, and *C*. *lindemuthianum* in leaves (Fig 2A). This was quite different for *PvLLG2*, where a high Z-scores (≥ 2) only was observed for *R*. *giardini* in roots, and no low Z-scores (≥ -2) were observed. Additionally, transcript accumulation of only *LLG1* was observed in *Glycine max* and *Medicago truncatula* during nodule development [42, 43] S2C and S2D Fig. By comparison, *PvLLG1* transcripts in this study detected using RT-qPCR were found to be highest in root hairs, followed by stems and root apex, and there was comparatively low expression in shaved roots, cotyledons, leaves, and flowers. *PvLLG2* transcript levels were only detectable in pollen, stigma and flower buds where levels were many times higher than those of *PvLLG1* Fig 2C.

**Fig 3:**
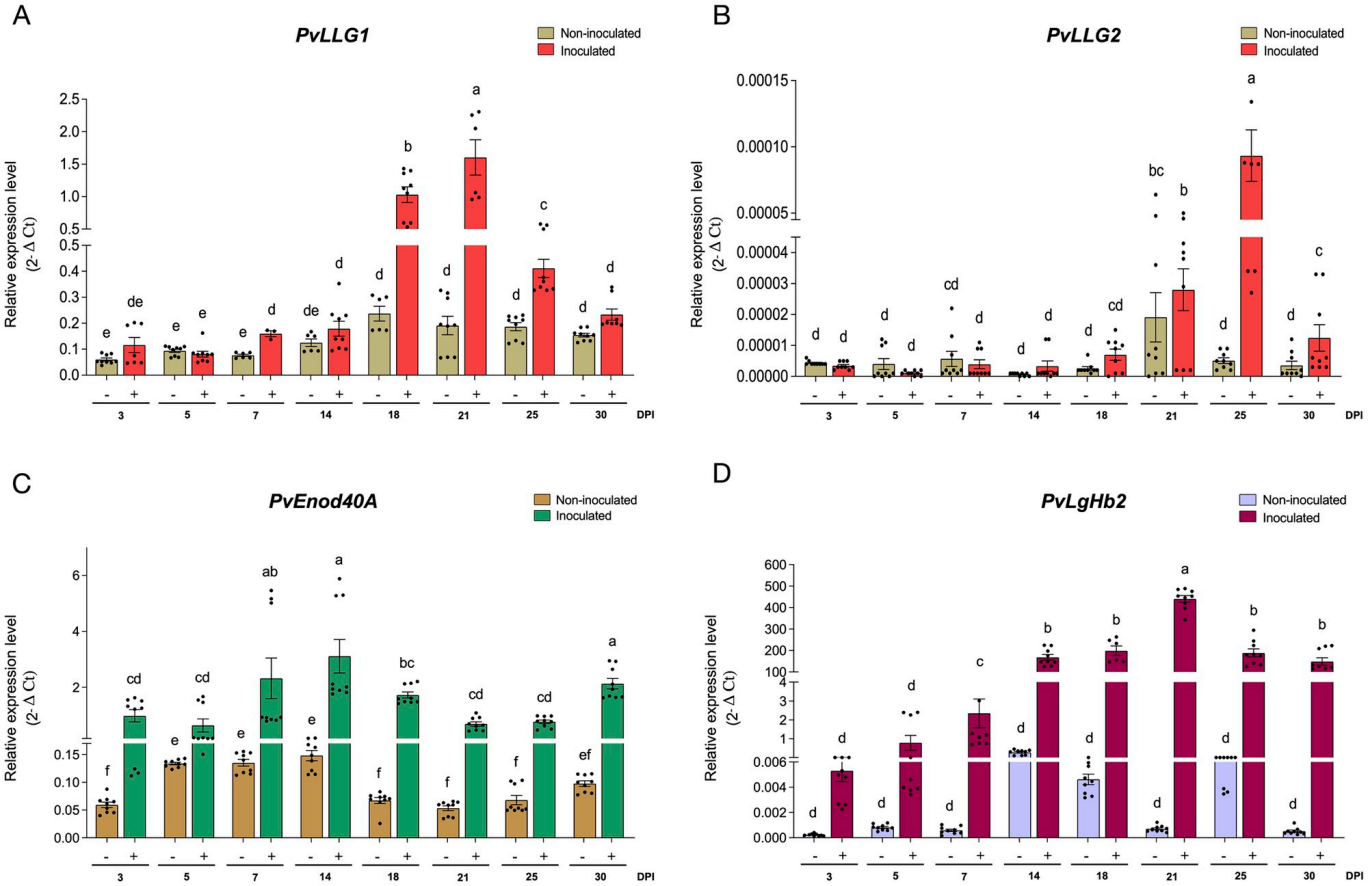
*PvLLG1* transcript accumulation during *R*. *tropici* interaction. **(A)** *PvLLG1*, (**B**) *PvLLG2*, (**C**) *PvEnod40*, *and* (**D**) *PvLgHb2* transcript accumulation in *P*. *vulgaris* roots inoculated (green, purple, and orange bars) and uninoculated (brown, violet and beige bars), at 3, 5-, 7-, 14-, 18-, 21-, 25- and 30- days post-germination. Quantification by RT-qPCR is normalized to *PvEf1α*. Bars represent ± SEM (Standard Error of Mean) of 2 biological replicates with 3 technical replicates each. Different letters indicate significant differences among samples according to the Two -Way ANOVA analysis at *p* <0.0001.

Transcript accumulation of *PvLLG1* following inoculation with *R*. *tropici*, which results in pink nitrogen fixing nodules first visible at 7 dpi and senescent by 30 dpi revealed a peak at 21 dpi, which was 12 times higher than expression when expression was first assessed at 3 dpi. Compared to the non-inoculated control, *PvLLG1* transcripts were significantly higher at 7, 18, 21 and 25 dpi (Fig 3A). *PvLLG1* transcripts in denodulated roots with *R*. *tropici* were much lower (5 to 10%) of that in isolated nodules, but both showed a significant increase between 14 and 21 dpi S3 Fig. *PvLLG1* transcripts in roots inoculated with *R*. *giardini*, which results in white non-nitrogen fixing nodules first visible at 7 dpi and senescent by 21 dpi, showed the highest expression at 7 dpi declining to 21 dpi. Compared to the non-inoculated control, *PvLLG1* transcripts were only significantly higher at 7 dpi and were significantly lower at 14 and 21 dpi (Fig 2D).

*PvLLG2* transcript accumulation showed a peak at 25 dpi with *R*. *tropici*, but the levels were always much lower than *PvLLG1* (max expression of 0.000093 versus 1.6) (Fig 3A). Compared to the non-inoculated control, *PvLLG2* transcripts were significantly higher at 25 and 30 dpi (Fig 3B). *PvLLG2* transcript accumulation with *R*. *giardini* showed a peak at 14 dpi, and expression was significantly higher than with the non-inoculated control at 7, 14 and 21 dpi (Fig 2D).

By comparison, *PvEnod40*, an early marker for nodule development, had significantly higher transcripts in *R*. *tropici* inoculated roots from 3 to 30 dpi compared to the non-inoculated control, with a peak in expression at 14 dpi (Fig 3C). Expression *PvLgHb2* a late molecular marker for nodule development was also significantly higher in *R*. *tropici* inoculated roots from 3 to 30 dpi compared to the non-inoculated control, with a peak at 21 dpi (Fig 3D).

### Promotor elements and activity of *PvLLG1* in roots

**Fig 4:**
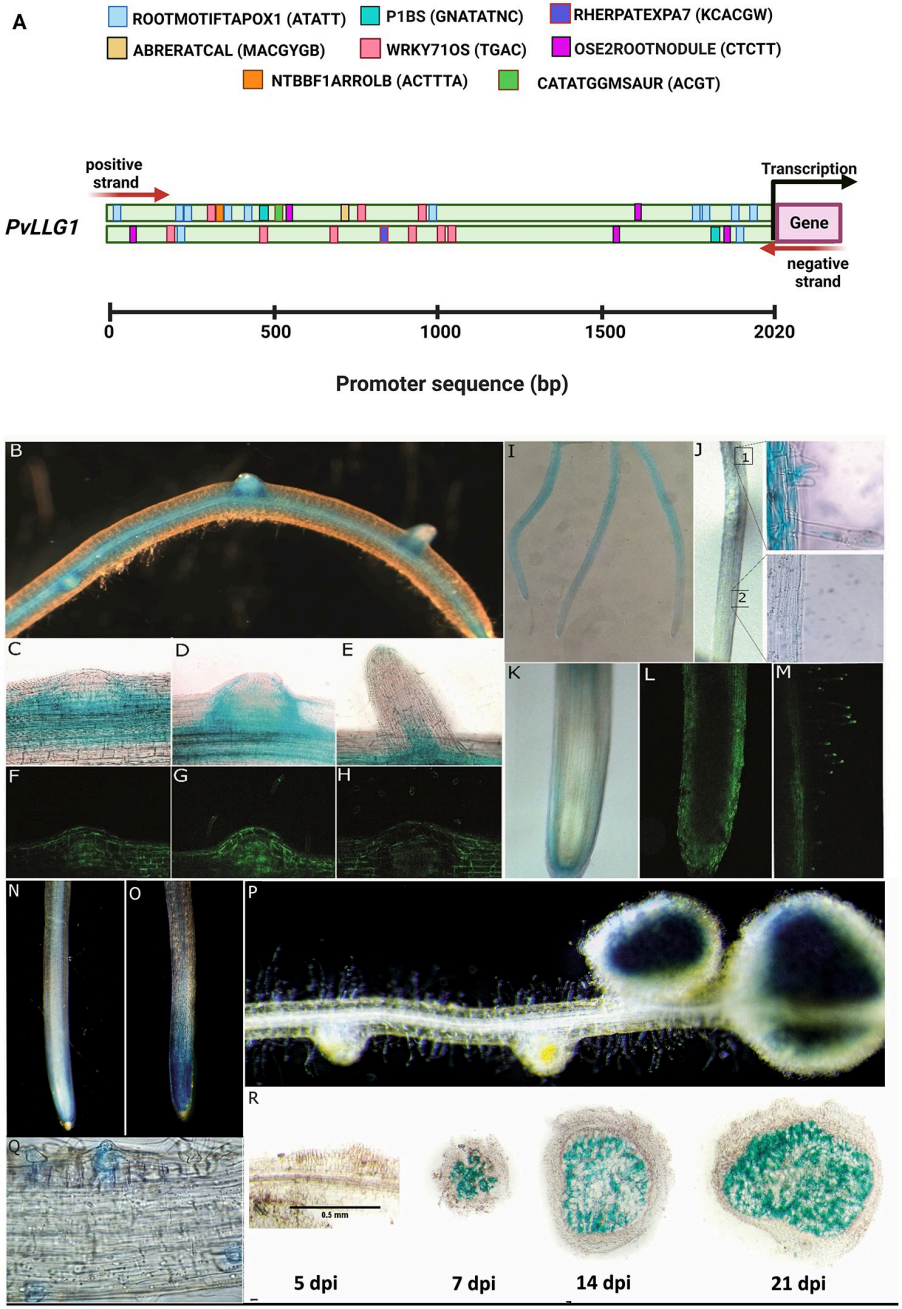
Analysis of *PvLLG1* promoter and expression in *P*. *vulgaris* roots and root hairs. **(A)** *PvLLG1* cis-regulatory elements in a promotor region of 2200 bp with ATATT (ROOTMOTIFTAPOX1) indicated with light blue boxes, CTCTT (OSE2ROOTNOULE) purple box, KCACGW (RHERPATEXPA7) violet blue boxes, GNATATNC (P1BS) magenta boxes, TGAC (WRKY71OS) pink boxes, ACGT (CATATGGMSAUR) green boxes, ACTTTA (NTBBF1ARROLB) ocher boxes, MACGYGB (ABRERATCAL) beige boxes. (**B-N**) *pPvLLG1*:*GFP-GUS* expression in non-inoculated *P*. *vulgaris* roots and nodules. (**B**) GUS in main and lateral roots, (**C**, **D** and **E**) GUS and (**F**, **G**, and **H**) GFP at different stages of a lateral root development, (**I and K**) GUS and (**L**) GFP at the root tip in the lateral root cap, (**J)** GUS and (**M**) GFP in root hair cells, (**N**) GUS in non-inoculated roots. (**O-R**) *pPvLLG1*:*GFP-GUS* expression in *R*. *tropici*-inoculated *P*. *vulgaris* roots. (**O**) GUS in inoculated roots at 3 dpi, (**P**) GUS in root nodules at 21 dpi, (**Q**) GUS in root hairs with infection threads at 4 dpi, and (**R**) cross sections of nodules at 5, 7, 14 and 21 dpi.

An examination of the 2020 bp upstream promoter region of *PvLLG1* showed four root-specific cis-regulatory elements. There was ROOTMOTIFTAPOX1 related to root-specific gene expression with 10 copies on the + strand and 2 copies on the—strand, OSE2ROOTNOULE related to nodule development with 2 copies on the + strand and 3 copies on the—strand, RHERPATEXPA7 related to root hair development with 2 copies on the + strand and 3 copies on the—strand, and P1PBS related to phosphate starvation response with 1 copy on the + strand and 1 copy on the—strand (Fig 4A). There were also two cis-regulatory elements involved in biotic and abiotic stress responses: WRKY71OS related to pathogen defense as well as senescence and trichome development with 3 copies on the + strand and 6 copies on the—strand, and CATATGGMSAUR related to dehydration heat and dark-induced senescence with 1 copy on the + strand and none on the—strand. Finally, there were two cis-regulatory elements involved in responses to plant hormones and other signaling molecules: NTBBF1ARROLB related to auxin responses and tissue-specific expression with 1 copy on the + strand and none on the—strand, and ABRERATCAL related to calcium response with 1 copy on the + strand and none on the—strand (Fig 4A).

A transcriptional fusion (*pPvLLG1*::*GUS-GFP*) was made with *GUS-GFP* and the 2022 bp of the *PvLLG1* promoter containing the cis-elements described above. In roots not inoculated with *R*. *tropici*, *PvLLG1* promoter activity was strong at the base of emerging lateral roots Fig 4B–4D, 4F and 4G, weak in the root primordia Fig 4E and 4H, strong in root cortex and vascular bundle before the elongation and meristematic region Fig 4I, 4K and 4L, strong in root hair from the differentiating region of the root Fig 4J and 4M, the lateral root cap Fig 4K and 4L, and was relatively weak in the root apex (Fig 4N). In roots inoculated with *R*. *tropici*, *PvLLG1* promoter activity was strong in the root apex at 5 dpi (Fig 4O), weak in roots at 5 dpi but strong in nodules at 7, 14 and 21 dpi Fig 4P and 4R, and strong in root hairs at 3 dpi when infection threads first appeared (Fig 4Q). Merged images of *R*. *tropici* expressing DS-RED showed that red fluorescence from *R*. *tropici* overlapped with green fluorescence directed by the *PvLLG1* promoter in infected root cells S4A Fig.

### Subcellular localization of PvLLG1 in *P*. *vulgaris* root and during Rhizobium inoculation

**Fig 5:**
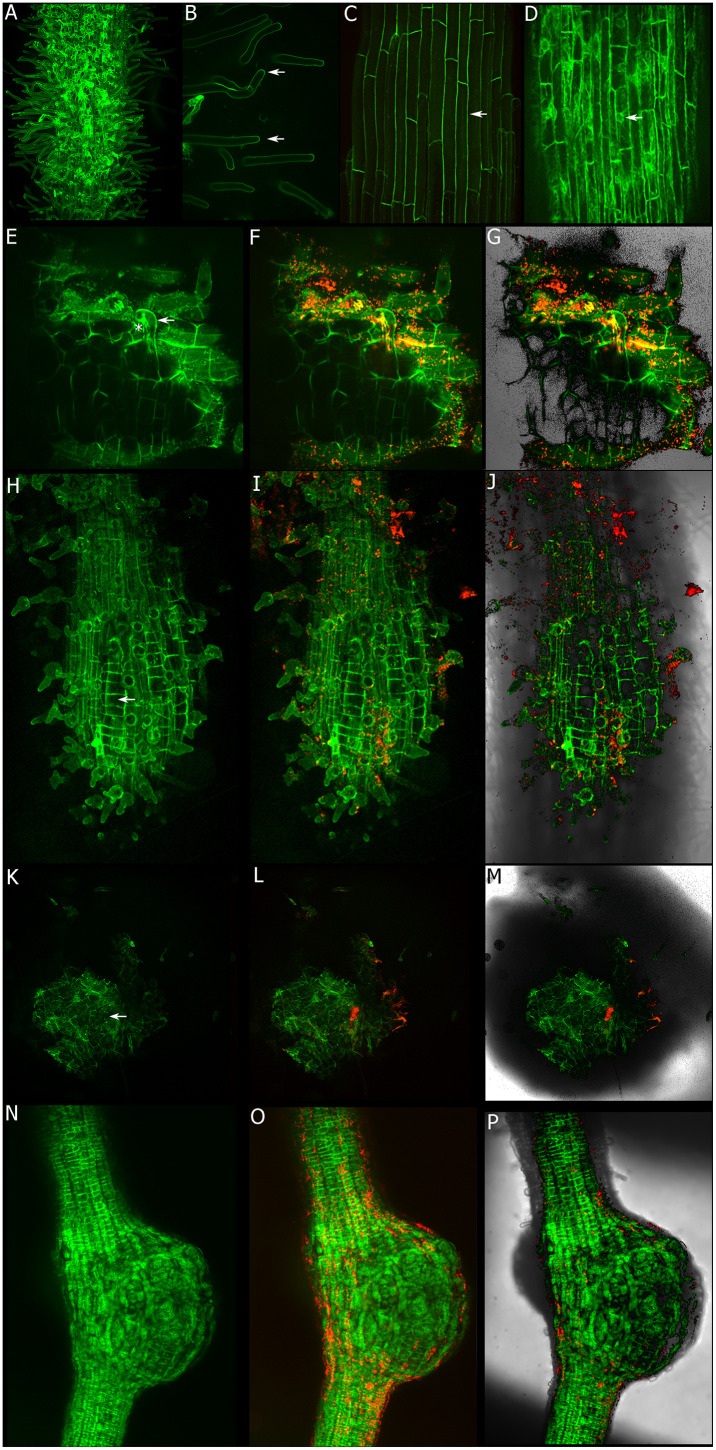
Subcelular localization of PvLLG1 under control and rhizobia inoculated roots. **A-C**) Subcelular localization of LLG1-Neon under its own promoter (*pLLG1*::*LLG1-Neon*). Note the plasma membrane localization in root hairs and cortical cells (see arrows). **D**) Subcellular localization of cytoplasmic GFP used as a control (see arrow). **E-G**) Subcellular localization during the infection thread formation (arrow). **H-J**) Subcellular localization during early cortical cell divisions induced by rhizobia inoculation (arrow). **K-M**) Subcellular localization in well-developed nodules (see arrow). **N-P**) Subcellular localization of cytoplasmic GFP in nodules used as a control.

Composite plants with transgenic roots expressing the endogenous promoter and the coding sequence fused to the gene of Neon (*pLLG1*::*PvLLG1-Neon*) were observed under confocal microscopy. As expected, the fusion protein is localized to the plasma membrane of root hairs and cortical cells Fig 5A–5C. This localization is very different to the control roots expressing the cytoplasmic GFP under the 35S promoter (Fig 5D). Rhizobia inoculated roots depict a clear increased of PvLLG1-Neon in root hairs forming the infection thread Fig 5E–5G and also in the cells starting the cell division that will form the nodule primordia Fig 5H–5J. Later on, during the nodule development there is a subcellular localization in the central part of the nodule forming the infected zone. This subcelular localization is very different to the control nodules expressing the cytoplasmic GFP Fig 5N–5P.

### Effect of silencing of *PvLLG1* on root hair length, and nodule development

**Fig 6:**
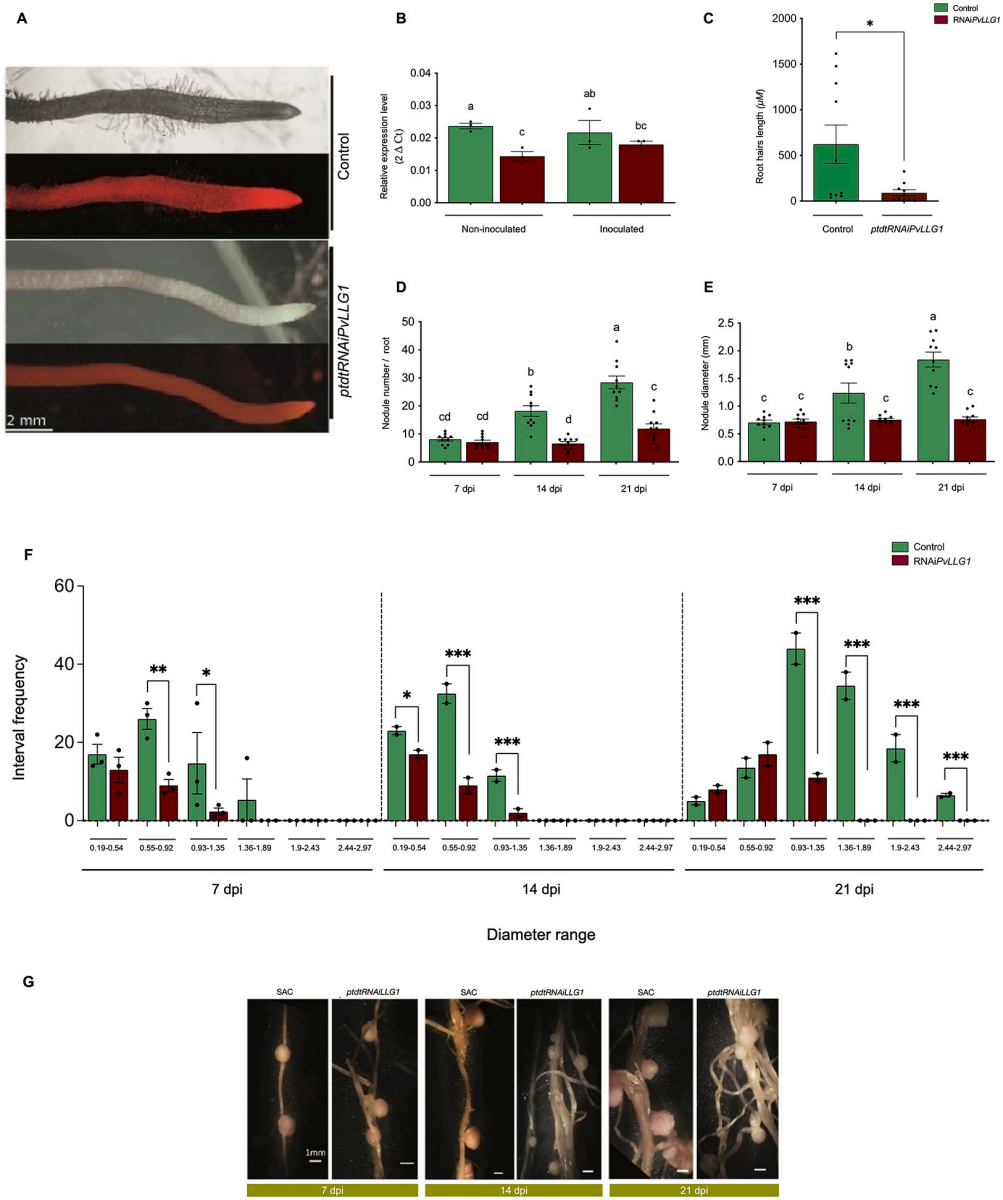
Effects of silencing *PvLLG1* on *P*. *vulgaris* in non-inoculated or *R*. *tropici*-inoculated roots. (**A**) Transgenic hairy roots showing expression of red fluorescent protein from nucleotide-scrambled sequence control (SAC) and *RNAi*:*PvLLG1* constructs. (**B**) *PvLLG1* transcript accumulation in *P*. *vulgaris* roots expressing SAC or *RNAi*:*PvLLG1* constructs under non-inoculated and *R*. *tropici*-inoculated condition. (**C**) Root hair length in *R*. *tropici*-inoculated roots with SAC or *RNAi*:*PvLLG1* constructs. (**D**) Nodule number at 7, 14, and 21 dpi in *R*. *tropici*-inoculated roots with SAC or *RNAi*:*PvLLG1* constructs. (**E**) Nodule diameter at 7, 14 and 21 dpi in *R*. *tropici*-inoculated roots with SAC or *RNAi*:*PvLLG1* constructs. (**F**) Number of nodules per composite plant with different ranges of diameters at 7, 14 and 21 dpi in *R*. *tropici*-inoculated roots with SAC or *RNAi*:*PvLLG1* constructs. (**G**) Images of root nodules from hairy roots expressing the SAC or *RNAi*:*PvLLG1* constructs at 7, 14 and 21 dpi in *R*. *tropici*-inoculated roots. Replications for *PvLLG1* transcript accumulation were 15, root hair length were 150, nodule number were 5, and nodule diameter were 150. Different letters or asterisk indicate significant differences among samples according to the Two -Way ANOVA analysis (*p = 0.05, ** p = 0.01, *** p = 0.001) or a pair-wise comparison with a Student´s T- test (p <0.0001), respectively.

Composite plants generated by *Agrobacterium rhizogenes* and expressing the silencing construct *pTDT-DC-RNAi* for *PvLLG1* revealed red fluorescence derived from the red fluorescent protein tdTomato, which was also observed for the control silencing construct *pTDT-Sac-RNAi* for a nucleotide-scrambled sequence (SAC) (Fig 6A). Examining the transformed hairy roots not inoculated with *R*. *tropici* revealed that *PvLLG1* transcript levels were significantly decreased (42%) at 10 d post-transformation compared to the SAC control (Fig 6B). In the transformed hairy roots inoculated with *R*. *tropici*, *PvLLG1* transcript levels were also significantly decreased (55%) by silencing compared to the SAC control (Fig 6B).

*PvLLG1* silencing significantly lowered root hair length by 80% in 5 day old roots comparison to the SAC control (Fig 6C). Furthermore, at 7 dpi there is no difference in the nodule number, however, at 14 and 21 dpi, silenced roots had 64% and 58% significantly reduced nodule number, respectively, compared to the SAC control (Fig 6D). Silencing also decreased nodule dry weight by 42% compared to the SAC control S5 Fig. Compared to the SAC control, silencing resulted in no significant differences in nodule diameter at 7, but at 14 and 21 dpi, nodule diameter was significantly decreased by 39% and 59% (Fig 6E). As silencing resulted in a wide range of nodule diameters, the number of nodules with different ranges of diameters were plotted. At 7 dpi, there was significantly difference in the nodule diameter ranges 0.19–0.54 mm and 0.55–0.92 of silenced plants with respect to controls (Fig 6F). However, the diameter ranges from 0.93–1.35 mm and 1.36–1.89 mm are more frequent for the WT than silenced plants. At 14 dpi, the number of nodules with relatively smaller diameter ranges (0.19–0.54, 0.55–0.92 mm) from silenced plants was also significantly more than the control. However, the diameter ranges from 0.93–1.35 mm are more frequent for the WT than the silenced condition. At 21 dpi, the number of nodules with larger diameter ranges (0.93–1.35, 1.36–1.89,1.90–2.43, 2.44–2.97 mm) from silenced plants were significantly less frequent than the control (Fig 6F). It is noteworthy to mention that diameters from 1.36 to 2.97 mm were only observed on the control and not in the silenced condition. Representative nodules are depicted in (Fig 6G).

### Effect of overexpression of *PvLLG1* on root hair length, and nodule development

**Fig 7:**
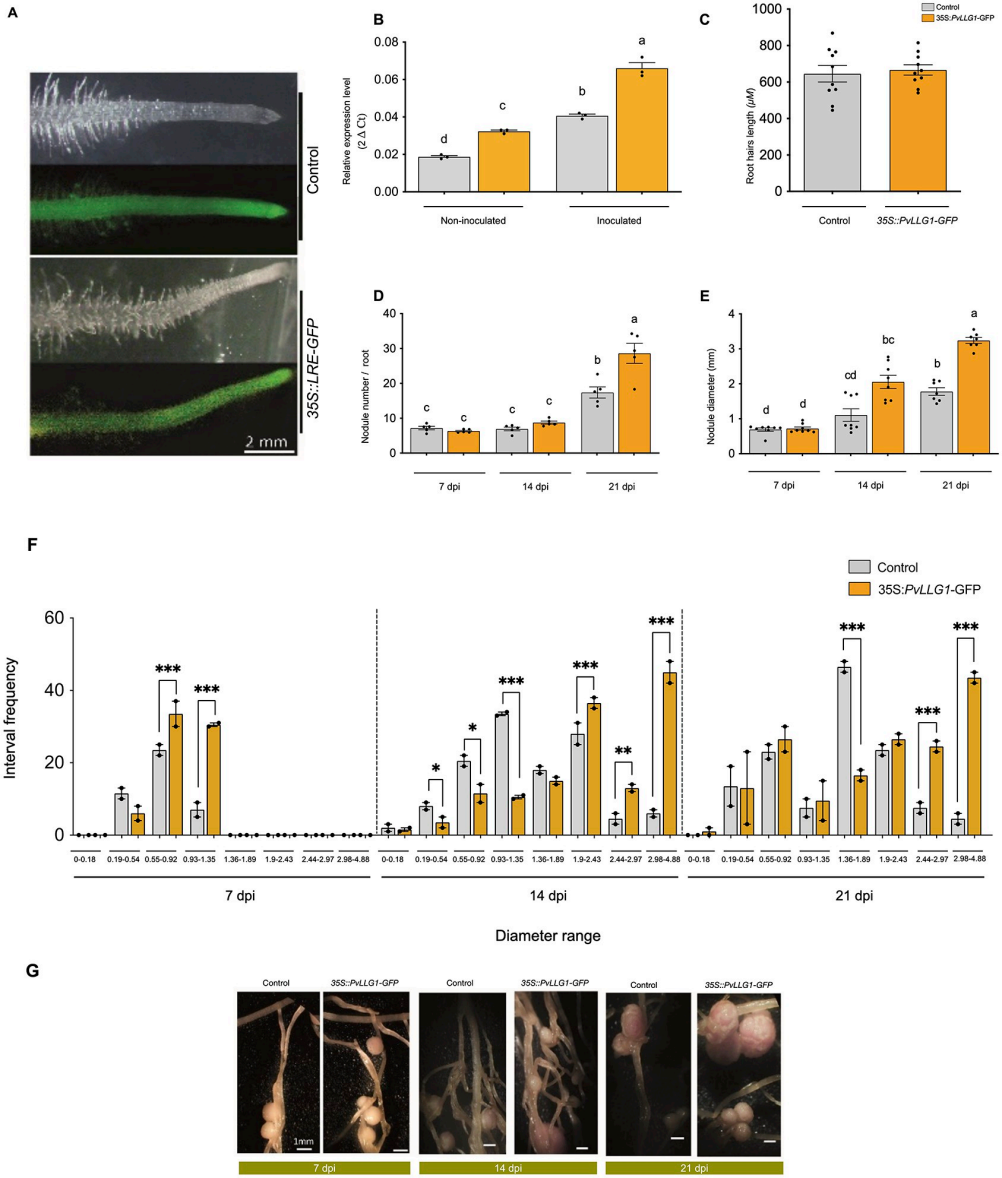
Effect of overexpression of *PvLLG1* in *P*. *vulgaris* for non-inoculated or *R*. *tropici*-inoculated roots. (**A**) Transgenic hairy roots show the expression of green fluorescent protein from the 35S::GFP (control) and *35S*:*PvLLG1-GFP* (overexpression) constructs. (**B**) *PvLLG1* transcript accumulation in non-inoculated and *R*. *tropici* -inoculated *P*. *vulgaris* roots expressing *35S*::*GFP* or *35S*:*PvLLG1-GFP*. (**C**) Root hair length in *R*. *tropici*-inoculated roots with *35S*::*GFP* and *35S*:*PvLLG1-GFP* constructs. (**D**) Nodule number at 7, 14, and 21 dpi in *R*. *tropici*-inoculated roots with *35S*::*GFP* and *35S*:*PvLLG1-GFP* constructs. (**E**) Nodule diameter at 7, 14 and 21 dpi in *R*. *tropici*-inoculated roots with *35S*::*GFP* or *35S*:*PvLLG1-GFP* constructs. (**F**) Number of nodules per composite plant with different ranges of diameters at 7, 14 and 21 dpi in *R*. *tropici*-inoculated roots with *35S*::*GFP* and *35S*:*PvLLG1-GFP* constructs. (**G**) Images of representative root nodules from hairy roots at 7, 14 and 21 dpi in *R*. *tropici*-inoculated roots with *35S*::*GFP* and *35S*:*PvLLG1-GFP* constructs. Replications for *PvLLG1* transcript accumulation were 15, root hair length were 150, nodule number were 5, and nodule diameter were 150. Different letters or asterisk indicate significant differences among samples according to the Two -Way ANOVA analysis (*p = 0.05, ** *p* = 0.01, *** *p* = 0.001) or a pair-wise comparison with a Student´s T- test (p <0.0001), respectively.

Composite plants with the overexpression vector *35S*::*PvLLG1-GFP* resulted in a strong green fluorescence from GFP indicating expression similar to that of a *35S*::*GFP* control (Fig 7A). Root tissues with *35S*::*PvLLG1-GFP* and not inoculated with *R*. *tropici* had an increase of 68% in its expression of *PvLLG1* compared to the *35S*::*GFP* control (Fig 7B). While the level of expression in *35S*::*PvLLG1-GFP* plants inoculated roots, the was an increase of 56% as compared to control roots due to induced expression by the infection (Fig 7B).

Overexpression of *PvLLG1* under non-inoculated condition resulted in no significant difference in root hair length compared to control plants (Fig 7C). Nodule number was not significantly different at 7 and 14 dpi after *R*. *tropici* inoculation with overexpression compared to the control (Fig 7D). However, there is a significant difference at 21 dpi, with increased nodule number of 33% in the overexpressing condition (Fig 7D). Nodule dry weights were significantly increased (44%) under overexpression conditions compared to the control S5 Fig. The nodule diameter was different at 14 dpi, with overexpressing plants presenting a bigger diameter. This difference in nodule diameter was increased with overexpression at 21 dpi (53%) (Fig 7E). Overexpression also resulted in a wide range of nodule diameters, and plotting the number of nodules with different diameters showed significantly more nodules at 0.55–0.92 and 0.93–1.35 mm at 7 dpi as compared to control (Fig 7F). This response is also observed at 14 dpi in root nodules overexpressing *PvLLG1*, which resulted in significantly more nodules with larger diameters 1.9–4.85 mm. Interestingly the control presents a higher frequency of nodules with small diameter mm at 14 dpi (0.19–1.35 mm). Finally at 21 dpi we observed that the nodules with larger diameters (2.44–4.85 mm) is maintained in the overexpressing lines. Representative nodules are depicted in (Fig 7G).

### Effect of silencing of *PvLLG1* on bacterial colonization, nitrogen fixation, superoxide and H_2_O_2_ levels

**Fig 8:**
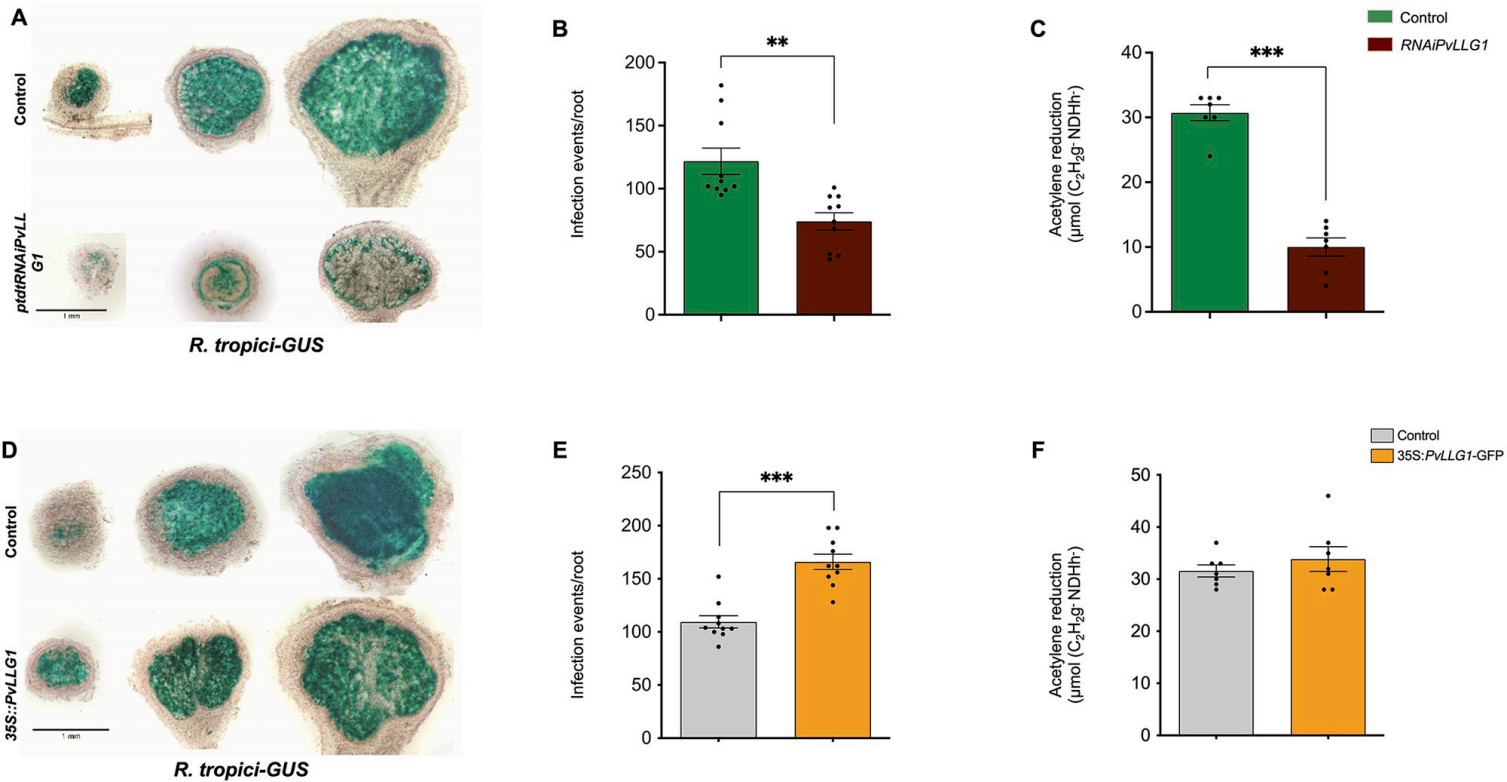
*Rhizobium tropici* colonization and nitrogen fixation in *PvLLG1* silenced and overexpressing *P*. *vulgaris* nodules. (**A**) Cross sections of hairy root nodules colonized by *R*. *tropici*-GUS showing bacterial colonization at 7, 14 and 21 dpi in *PvLLG1* silenced plants (*RNAi*:*PvLLG1* construct) compared to control (SAC). (**B**) Infection thread formation at 7 dpi in *PvLLG1* silenced plants compared to control. (**C**) Nitrogen fixation (acetylene reduction) at 21 dpi in *PvLLG1* silenced plants compared to control. (**D**) Cross section of nodules from hairy root colonized by *R*. *tropici*-GFP showing bacterial colonization at 7, 14 and 21 dpi in *PvLLG1* overexpressing plants (*35S*:*PvLLG1-GFP* construct) compared to control (*35S*::*GFP* construct). (**E**) Infection thread formation at 7 dpi in *PvLLG1* overexpressing plants compared to control. (**F**) Nitrogen fixation (acetylene reduction) at 21 dpi in *PvLLG1* overexpressing plants compared to control. Asterisks indicate significant differences among samples according to the Two -Way ANOVA analysis (*p = 0.05, ** *p* = 0.01, *** *p* = 0.001).

**Fig 9:**
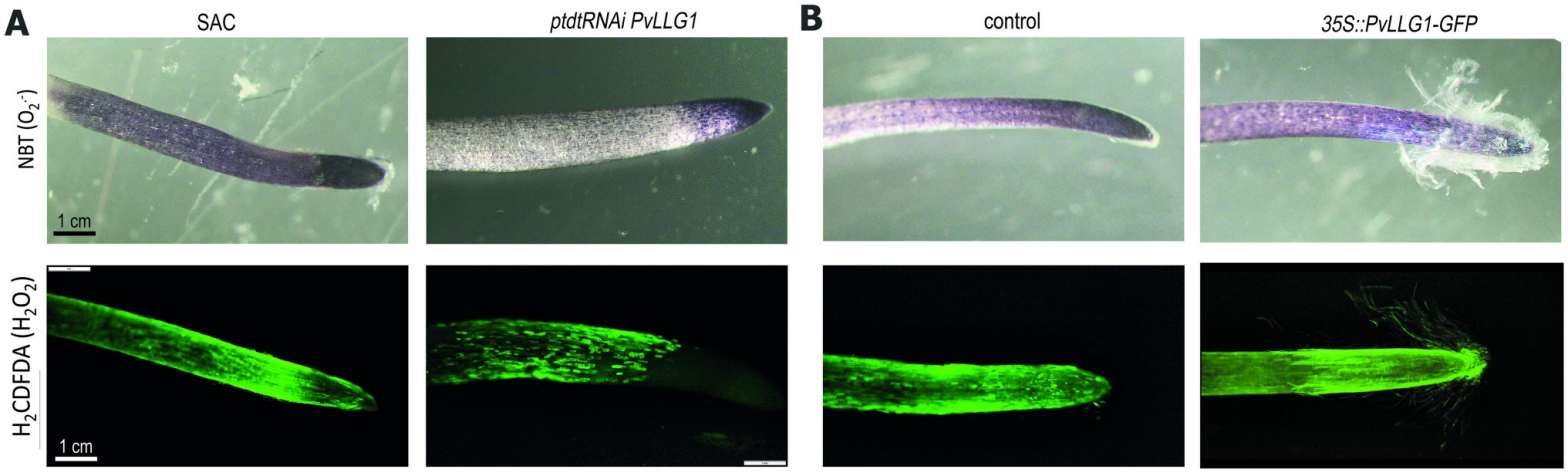
ROS distribution in *PvLLG1* silenced and overexpressing *P*. *vulgaris* roots. (**A**) Control (SAC) and *PvLLG1* silenced (*RNAi*:*PvLLG1* construct) roots with NBT staining to indicate superoxide distribution (upper panel), and H_2_CDFDA fluorescent probe to indicate ROS distribution in roots (middle panel), and epidermal zone where root hairs emerge (bottom panel). (**B**) Control (*35S*::*GFP*) and *PVLLG1* overexpressing (*35S*:*PvLLG1-GFP* construct) roots with superoxide distribution (upper panel), ROS distribution (middle panel) and ROS distribution in emerging root hair cells (bottom panel).

In *PvLLG1* silenced nodules at 7, 14 and 21 dpi, there was less *R*. *tropici-GUS* colonization compared to the control (Fig 8A). The smaller nodules in silenced plants also showed reduced peripherical rhizobial colonization compared to the control with most of the rhizobia located in the outer cell layers of the nodule, leaving large areas without colonization compared to a more uniform colonization in the control. Similar results were obtained with colonization by *R*. *tropici*-GFP S4B Fig. Silencing *PvLLG1* significantly decreased the number of infection threads (Fig 8B). Nitrogen fixation was also significantly reduced with *PvLLG1* silencing compared to the control (Fig 8C). Silencing *PvLLG1* resulted in less superoxide at the tip and in the elongation zone, as indicated by a redistribution of NBT staining Fig 9A upper panel and S6A and S6B Fig. Silencing *PvLLG1* also resulted in a reduced H_2_O_2_ signal at the meristematic region and a spotted pattern of H_2_O_2_ along the elongation zone as compared to the control as indicated by H2CDFDA staining Fig 9A lower panel and S6C and S6D Fig. Root hairs appeared reduced in length and number as compared to the control S6E and S6F Fig.

### Effect of overexpression of *PvLLG1* on bacterial colonization and nitrogen fixation, superoxide and H_2_O_2_ levels

Overexpressing *PvLLG1* resulted in greater colonization of the nodule by *R*. *tropici-GUS* at 7 dpi but similar colonization at 14 and 21 dpi compared to the control (Fig 8D). The results were the same with colonization by *R*. *tropici-DsRED* S4C Fig. Overexpression significantly increased the number of infection threads (Fig 8E). However, *PvLLG1* overexpression did not significantly change nitrogen fixation compared to the control (Fig 8F). Overexpressing roots had superoxide distributed from the apex to beyond the meristem region which is the elongating region, unlike control roots where it was limited to the apical meristem and a faint superoxide is observed in the elongating region Fig 9B, upper panel and S6G and S6H Fig. *PvLLG1* overexpression resulted in H_2_O_2_ that was similarly distributed in the elongation zone as the control Fig 9B, right bottom and S6I and S6J Fig. However, root hairs appeared to have been normally developed as in the control S6K and S6L Fig.

## Discussion

Two *LLG* genes (*PvLLG1* and *PvLLG2*) were found in the *P*. *vulgaris* genome, which were closely related to *AtLLG1* and *AtLLG2*. In addition, there were homologs of *LLG1* and *LLG2* in the genomes of seven other legume species. However, none of the *LLG* genes from legumes had higher identity to *AtLLRE* than *AtLLG1*, or *AtLLG3* than *AtLLG2*, and thus it appears that legumes only have homologs of *AtLLG1* and *AtLLG2*. All the legume species examined had only one *LLG1* and *LLG2* gene, except for *G*. *max* that had three *LLG1* and two *LLG2* genes, *A*. *hypogea* that had four *LLG2* genes and *M*. *truncatula* that had two *LLG2* genes. This may have been due to relatively recent gene duplications followed by divergence over time. There is evidence of polyploidy in *G*. *max*, *A*. *hypogea*, and *M*. *truncatula* [44, 45], and thus entire genome duplications could be related to the multiple variants of *LLG1* and *LLG2* genes. One possibility is that multiple *LLG* genes provide a stronger response, but it could also allow for specialization of the response to differentiate signals. The binding of LLGs with RALF23 peptides nucleates the assembly of RALF23-LLG1- FER and RALF23-LLG2-FER heterocomplexes, which results in the modulation of ROS production in a NADPH-dependent manner (Xiao et al., 2019). LLGs also bind FER as a cochaperone regulating the movement of CrRLK-FER from the ER to the plasma membrane to allow FER to function in the cell signal transduction by modulating the proton H^+^ AHA pump [46]. Thus, variants of LLGs could affect both ROS production and microbe recognition.

Several highly conserved regions of LLG proteins have been identified based on the LLG sequences in *A*. *thaliana* [28, 31]. Two of these are the 13 amino acids for RLF23 binding and the 8 amino acid motif (KEGKEGLE/D) for complementing the RLF23 binding. However, this study showed that those amino acids are not all highly conserved in the legume plant family, particularly for the KEGKEGLE/D motif. Thus, it appears that there is a broader range of amino acids possible for those functions. An examination of LLG sequences from a very broad range of plants in many plant families is needed to better understand the range of variation possible for those regions. Predicted protein models of the *P*. *vulgaris* and *A*. *thaliana* LLG1s and LLG2s indicated that such differences affected the protein surface, and thus may affect their ability to bind to ligands [28]. Binding studies, however, are needed to confirm if the differences result in any alteration of the predicted LLG proteins to interact with RALF23, FER, or CrRLK.

Surprisingly, *GmLLG1-3* from *G*. *max* was predicted to lack a hydrophobic tail. The hydrophobic tail of LLG proteins is required for it to processed by GPI transamidase for plasma membrane localization [47]. However, it has been reported that lack of post translational GPI modification might not affect the plasma membrane localization [31]. Thus, the lack of the hydrophobic tail does not necessarily denote that the GmLLG1-3 protein is not localized to the plasma membrane. However, *G*. *max* has three variants of LLG1, and one possibility is that the lack a hydrophobic tail in one of them could be related to specialization among LLG1s for interaction with ligands other than RALF23 or FER.

*PvLLG1* was highly expressed in vegetative tissue, while *PvLLG2* was highly expressed in floral tissue and very low in vegetative tissue. In Arabidopsis, *AtLLG1* is strongly expressed in vegetative tissues, *AtLLG2* and *AtLLG3* are expressed in reproductive tissues, and *AtLRE* is expressed only in synergistic cells [17, 25, 27]. Thus, expression of the LLG1 and LLG2 genes of *P*. *vulgaris* and *A*. *thaliana* are very similar, indicating similar roles. Differential expression of LLG proteins and their interaction with its tissue-specific ligands have been proposed to determine the wide variety of responses involving CrRLK receptors [24].

LLGs may play an important role in plant-microbe interactions. The binding of FER to AtLLG1 affects FER’s ability to determine the subcellular location of the PAMP receptor FLG22 [21, 23, 28, 48, 49]. Mutants of *AtLLG1* showed enhanced susceptibility to pathogens supporting an important role in pathogen recognition [49]. This role in recognition of PAMPs of plant pathogens could explain the Phytozome database showing *PvLLG1* induction in leaves by *P*. *phaseolicola* infection, as well as *PvLLG2* induction by *R*. *giardini* infection of roots, which does not result in nodule formation.

Since plant pathogenic and mutualistic interactions have many similarities [50], LLG proteins may also regulate mutualistic interactions. This would be consistent with the results in the Phytozome database showing increase expression of *PvLLG1* in roots with the mutualistic interactions with *R*. *tropici* and *R*. *irregularis*. One shared feature of rhizobial nodule formation and pathogen infection is that they are both ROS-regulated processes, which could involve LLG proteins acting as mediators [32–35]. LLG proteins could be directly important for those ROS-regulated processes as LLG binds with FER, FER activates GEF and RAC/ROP GTPase which recruit and activates RboH resulting in ROS production [13, 51, 52]. RALF/FER together with LLG partially targets the PTI-related FLS2/BAK1 complex to modulate ROS levels, which allows for recruitment of beneficial microbes by sensing plant cell wall integrity [51, 53–55].

Initiation of the infection thread by *R*. *tropici* and early host cell divisions in the root outer cortical cells was observed within the first 3 dpi. Although there was no significant increase in *PvLLG1* transcript accumulation at 3 dpi, promotor activity of *PvLLG1* was detectable in inoculated roots in root hairs corresponding with the appearance of infection threads and then was detected at the root apex at 5 dpi. This promoter activity correlates with the increased accumulation of the PvLLG1-Neon in the infection thread. *PvLLG1* promoter activity and subcelular localization of PvLLG1-Neon in the early infection thread suggests similar role as in polar tip growth. Polar growth occurs in legume root hairs during infection thread migration, which could require ROS from NADPH oxidase activity in a FER-LLG-ROP dependent manner as described for Arabidopsis root hair growth [13]. Infection thread formation and the first cell divisions beneath this structure are two different and independent processes, but both require extensive cell wall remodeling, including pH, ROS and Ca^2+^ changes as well as cytoskeletal rearrangements [2, 4]. Infection thread formation also requires cell wall reorganization, and PvLLG1 interaction with FER could also be important at this stage of the infection as FER is involved in maintaining de-esterified pectin, a key component of infection threads [56]. Therefore, the reduced root hair lengths observed in *PvLLG1*-silenced plants could account for the reduced infection thread phenotype and nodule number.

By 5 dpi, nodule primordia were small, but could be visualized in roots. However, these structures did not show *PvLLG1* promoter activity. At 5 dpi, *PvLLG1* silenced plants had less superoxide at the root tip and elongation zone, and spotty H_2_O_2_ in the elongation root zone. They also had significantly reduced root hair length. However, none of those were affected in *PvLLG1* overexpression plants, indicating that silencing had a much greater effect on the interaction than overexpression. In Arabidopsis, LLG1 can bind FER to regulate cell growth and development of root hairs as well as pollen-stigma interaction [17, 57], and therefore, mutations in *AtLLG1* showed impaired root hair development related to impaired ROS production [49]. The finding that silencing *PvLLG1* reduced root hair length indicates that its role in root hair development is very similar to that described in Arabidopsis in accordance with a role for LLGs in ROS generation by NADPH oxidases [17, 58].

At 7 dpi, more developed nodule primordia were observed with the infection thread continuing to grow through the cortical cells to reach the inner cells. At this stage of development, clear *PvLLG1* promoter activity was observed in the nodule and significant transcript accumulation was detectable. *PvLLG1* silencing reduced *R*. *tropici* colonization, reduced the number of infection threads, but did not affect nodule diameter. *PvLLG1* overexpression increased *R*. *tropici* colonization, increased the number of infection threads, nodule number and nodule diameter. The finding that silencing *PvLLG1* had a negative effect on rhizobial colonization and nodule development, while overexpression stimulates these responses both suggests that *PvLLG1* has a pivotal role at this stage of the interaction.

By 14 dpi, nodules were completely formed, and infected cells harbor differentiated bacteroids, corresponding to the start of nitrogen fixation. At this time there was clear expression of the *PvLLG1* promoter activity throughout the nodule. *PvLLG1* silencing reduced *R*. *tropici* colonization, nitrogen fixation and nodule diameter, while *PvLLG1* overexpression increased rhizobial colonization and nodule diameter but it did not affect nitrogen fixation. Again, this suggests that *PvLLG1* silencing more impacted the interaction than overexpression.

By 21 dpi, nodule size and nitrogen fixation were at their maxima. *PvLLG1* promoter activity had increased, and its transcript accumulation had reached its maximum. *PvLLG1* silencing resulted in reduced *R*. *tropici* colonization, nodule number, nodule diameter and nitrogen fixation. On the other hand, *PvLLG1* overexpression resulted in increased *R*. *tropici* colonization and nodule diameter, although nodule number and nitrogen fixation were not affected. The increased nodule diameter under *PvLLG1* overexpression could be related to higher levels of ROS on the plant cell walls, allowing them to expand. It is well established that ROS affects plant cell development [49, 59–61].

Finally by 30 dpi, nodules were senescent and had stopped fixing nitrogen. *PvLLG1* promoter activity and transcript accumulation decreased, and there was also decreased transcript accumulation of *PvLgHB2*. The Leg hemoglobin gene is important for generating the low oxygen conditions required for rhizobia nitrogenase activity and declines when its role in nitrogen fixation is no longer required, such as during nodule senescence [62]. Therefore, *PvLLG1* promoter activity and transcript accumulation seem to correlate with both the degree of rhizobial colonization and their activity (i.e., nitrogen fixation).

In non-inoculated roots, clear *PvLLG1* promoter activity was observed at the base of the emerging lateral root, but not in the meristematic region. This is very similar to 5 dpi of *R*. *tropici* inoculated roots for young primordia, and suggests that *PvLLG1* could have a negative role in regulating both lateral root primordia and early nodule primordia. Therefore, *PvLLG1* could be part of a temporal up-regulation of ROS to facilitate the onset of the meristem, and then decreased *PvLLG1* transcript accumulation could be related with the end of the transient ROS change. Since meristem development is highly dependent on ROS [61, 63], it will very interesting to assess the potential role of *PvLLG1* during lateral root development.

Although transcript accumulation of *PvLLG1* was highly correlated with nodule formation, *PvLLG2* transcript accumulation was also detected during nodule formation but at much lower expression values. Likewise, both *PvLLG1* and *PvLLG2* expression peaked at 21 dpi, showing similar temporal patterns. However, *PvLLG1* showed greater expression during the nitrogen fixation stage (18–21 dpi) when inoculated with *R*. *tropici*, whereas *PvLLG2* showed greater expression with *R*. *giardini* inoculation. Since *R*. *giardini* is a nod+ but Fix- species, it is possible that *P*. *vulgaris* expressed high levels of *PvLLG2* to reject the bacterial colonization, as *R*. *giardini* is more like a pathogen obtaining plant nutrients but not providing benefits with an empty nodule phenotype [64]. So far, this is the only data showing that a *LLG2* gene can be expressed in vegetative tissue, and indicates that it may have an unexpected role in plant-microbe interactions. Since pH, ROS and Ca^2+^ are highly dependent from CrRLK receptors, it remains to be determined if *LLG2* genes can orchestrate pH, ROS and Ca^2+^ changes to prevent infections by non-mutualistic microbes in a similar way that *LLG1* genes appear to be able to coordinate root hair polar growth and infection tread formation during a mutualistic rhizobial interaction. Furthermore, it has been recently described that PvFER1, PvRALF1, and PvRALF6 regulate the optimal number of nodules as a function of nitrate availability, and therefore, PvLLG1 could also be an important modulator of that response [65].

In conclusion, legumes appear to have only homologs of *LLG1* and *LLG2* genes, although some species have multiple variants of each. For *P*. *vulgaris*, there was one *LLG1* and *LLG2* gene that were expressed in different tissues. However, both genes were expressed during nodule development peaking with mature nodule formation, although *PvLLG1* expression was much greater and correlated with the onset of nitrogen fixation. *PvLLG1* silencing and overexpression generally had opposite effects on the interaction, although more factors were significantly affected by silencing. The results indicate that *PvLLG1* may be required during the early stages of infection (3–5 dpi) to avoid an immune response, perhaps by affecting PAMP receptors, allowing rhizobial colonization and infection thread formation. However, *PvLLG1* is also important later in the interaction in infected cells probably restricting over-colonization due to the regulation of the production and distribution of ROS in roots. One challenge in the study of rhizobia is to understand how plants can recruit them and restrict pathogens at the same time. There are models about how plants select for rhizobia [66], but how this differs with non-mutualists, such as *R*. *giardini*, is poorly understood. While this study has demonstrated a role for a *LLG1* gene in a mutualistic microbe-root interaction, in the future, it would be worthwhile to investigate the role of *LLG1* and *LLG2* genes in a broader variety of microbe-root interactions.

## Materials and methods

### Phylogenetic analysis

For phylogenetic analysis, the amino acid sequences of AtLRE, AtLLG1, AtLLG2, and AtLLG3were used as queries reference to identify LRE/LLG sequences of in seven species of the Fabaceae family, *P*. *vulgaris*, *L*. *japonicus*, *G*, *max*, *M*. *truncatula*, *L*. *culinaris*, *C*. *arietinum* and *A*. *hypogea* (S1 and S2 Tables). All sequences were downloaded from the Phytozome database v. 13 (https://phytozome-next.jgi.doe.gov). Alignment of the amino acid sequences was performed with MUSCLE in JALVIEW and EMBOSS (https://www.ebi.ac.uk/Tools/emboss/). Characteristic domains and motifs of LRE/LLG sequences were identified in MEME Suite (https://meme-suite.org/meme/) and InterPro-Pfam (https://www.ebi.ac.uk/interpro/). Analysis of phylogenetic relationships between sequences was performed with IQ-TREE (http://iqtree.cibiv.univie.ac.at) using Maximum Likelihood method with 1,000 bootstraps. The consensus tree was edited with iTOL (https://itol.embl.de). Tertiary structures based on the amino acid sequences were analyzed in SWISS MODEL (https://swissmodel.expasy.org). Structures were downloaded in PDB format from SWISS MODEL, and the best fit of the tertiary projection of the protein was made. The PDB file was analyzed in PyMOL (https://pymol.org/2/) to provide a globular model of the predicted proteins. The projection angle of the proteins was 45° front-upper. PyMOL was also used to highlight the amino acids involved in binding with RALF23 in red, the conserved motif for complementary binding with RALF23 in gray, and the amino acids common between first and second motif for binding with RALF23 in purple.

### *In silico* analysis

*PvLLG1* and *PvLLG2* transcriptional profiles were obtained from the Phytozome database v. 13, as well as profiles from Oblessuc et al. (2022) for *Pseudomonas phaseolicola*, Alvarez-Diaz et al. (2022) for *Colletotrichum lindemuthianum*, Khankhum et al. (2016) for *Edornavirus*, and Chen et al. (2020) for *Fusarium solani*. To identify the genes, these were aligned with the reference genome of *Phaseolus vulgaris* v 2.1 (Phytozome v. 13), with Bowtie2 v2.5.1. Total transcript counts within each condition were performed with eXpress v1.5.1. Differential expression analysis was carried out on the IDEAMex web server (Integrative Differential Expression Analysis for Multiple Experiments; Jiménez-Jacinto et al, 2019) of the University Unit of Mass Sequencing and Bioinformatics of the Biotechnology Institute-UNAM [67]. Differential expression analysis was performed with DESeq, EdgeR, NOISeq and Limma statistical methods included in the Bioconductor platform package. The z-scores were calculated with scripts designed in R. Statistical parameters were *p>* 0.05, FDR = 0.05 and CPM = 1 to determine the differential expression of the transcripts of each tissue and stage of development. Heatmaps graphics were designed in R.

### Vector construction and composite plants

In order to predict the c*is-acting* elements in the promoter of *PvLLG1* gene, 2022 bp upstream of the initiation codon (ATG) were downloaded from the Phytozome v.13 database. The cis-elements were predicted using PlantCare (http://bioinformatics.psb.ugent.be/webtools/ plantcare/html/). To create a transcriptional fusion construct to determine *PvLLG1* promoter activity, the 2022 bp sequence was amplified by PCR using *P*. *vulgaris* cv Negro Jamapa genomic DNA as template with gene-specific primers (S3 Table). The PCR product was cloned into the entry pENTR/D-TOPO vector according to the manufacturer’s instructions (Invitrogen, Waltham, Massachusetts, USA) and inserted into the destiny vector pBGWFS7, which has the GUS-GFP reporter genes fusion without promoter. The Gateway LR reaction was performed between the entry vector (pENTR/D-TOPO 2022pb *PvLLG1*) and pBGWFS7 according to the manufacturer’s instructions (Invitrogen). The transcriptional fusion construct was named *pPvLLG1*::*GFP-GUS*. An empty pBGWFS7 vector was used as the control in the transcriptional fusion experiments. The presence of the insert was confirmed by PCR and sequencing.

To make a *PvLLG1* silencing RNAi construct, a 224 bp fragment corresponding to the 3′-untranslated regions of the *PvLLG1* coding sequence was amplified from *P*. *vulgaris* cv Negro Jamapa cDNA from roots using gene-specific primers (S3 Table). The resulting PCR product was cloned into pENTR/D-TOPO vector (Invitrogen), and transferred to the destination vector *pTdT DC-RNAi* using the LR clonase of the Gateway system (Invitrogen) and according to the manufacturer’s instructions. The appropriate orientation of the insert was confirmed by PCR and sequencing. As control, a truncated unrelated sequence from Arabidopsis was used lacking the target sequence of miR159 (ACAGTTTGCTTATGTCGGATCCATAATATATTTGACAAGATACTTTGTTTTTCGATAGATCTTGATCTGACGATGGAAGTAGAGCTCTACATCCCGGGTCA), which was cloned into the *pTdT DC-RNAi* vector.

To design the construct for overexpressing *PvLLG1*, its open reading frame was amplified from *P*. *vulgaris* cv Negro Jamapa cDNA from roots using PCR with gene-specific primers S3 Table and cloned into the pENTR/D-TOPO. The Gateway LR reaction was performed between an entry vector (pENTR/D-TOPO containing the cloned *PvLLG1*) and the pH7WG2 binary vector under the control of the constitutive 35S promoter according to the manufacturer’s instructions (Invitrogen). An empty pH7WG2 vector, which constitutively expresses GFP, was used as the control. Again, the presence of the *LLG* gene was confirmed by Sanger sequencing and PCR.

The PvLLG1-Neon fusion was designed with its own promoter. The construct *pLLG1*::*PvLLG1-Neon* was synthetized by GenScript (New Jersey, USA) which contained 2018 pb from the PvLLG1 promoter region, the coding region for PvLLG1 and the gene encoding the fluorescent protein Neon. After the Neon sequence we included the GPI binding domain, the omega site for processing and the hydrophobic tail. This allow the PvLLG1-Neon to be modified with GPI as previously described [31].

All plasmid constructs were introduced by electroporation into *A*. *rhizogenes* strain K599 and used to generate composite *P*. *vulgaris* cv. Negro Jamapa plants as described (Estrada-Navarrete et al., 2007). Transgenic composite plants were observed under epifluorescence microscopy to confirm the presence of the reporter gene (GFP or DsRed), and untransformed roots were removed so that only roots expressing the transgene of interest were studied.

### Seed germination

Seeds of *P*. *vulgaris* L. cv. Negro Jamapa were surface sterilized with sodium hypochlorite (25%) for 5 min, rinsed five times with sterile water, incubated in pure ethanol for 1 min, and rinsed again for another five times with water [68]. Sterile bean seeds were transferred to sterile steel plates lined with wet paper towels with Fahraeus nutrient solution [69]. Plates were covered with Al foil, and incubated at 28°C for 2 d in the dark.

### Rhizobia inoculation

*Rhizobium tropici* CIAT899 was grown in 250mL flasks containing 100 mL of PY broth supplemented with 7 mM CaCl2, 50 μg mL^−1^ rifampicin, and 20 μg mL^−1^ nalidixic acid. The culture was incubated at 30°C and 250 rpm until it reached an OD600 of 0.8. For the nodulation assay, composite common bean plants were planted in pots with vermiculite previously sterilized, and inoculated with 1 mL of *R*. *tropici* suspension diluted to an OD600 of 0.05 in 10 mM MgSO_4_. Plants were grown in a controlled environment chamber (16 h light/8 h darkness, at 26°C) and watered twice with Fahraeus nutrient solution without nitrate potassium concentration. At 7,14, and 21 days after inoculation (dpi), nodules number, diameter of nodules and dry weight were determined. Ten plants per treatment (non-inoculated and inoculated with *R*. *tropici)* were evaluated, and two independent experiments were performed.

### Nodule diameter

Roots expressing the SAC, *RNAi*:*PvLLG1*, *35S*:*GFP*, and 3*5S*:*PvLLG1-GFP* constructs were inoculated with *R*. *tropici*-GUS and collected at 7, 14, and 21dpi. GUS staining was performed [70], and the roots were placed in a 140 x 20 mm Petri dish to be scanned under a Nikon Eclipse microscope (Nikon, Melville, NY, USA), using ImageJ software *v2*.*9* to determine the number and diameter of the nodules.

### Acetylene reduction assay

Acetylene reduction was used to indirectly quantify the nitrogenase activity in transgenic nodules at 21 dpi. Nodulated plant were transferred to 100 mL vials with rubber seal stoppers by injecting acetylene to a final concentration of 2% of the gas phase. Each sample was incubated for 60 min at room temperature, and ethylene production was determined by gas chromatography (Variant, model 3300) as previously described [71]. Specific activity was expressed as μmol ethylene g nodule dry weight^−1^h^−1^.

### Promoter activity analysis

Composite *P*. *vulgaris* plants harboring the transcriptional fusion *pPvLLG1*::*GFP-GUS* were collected at 3, 5, 7, 24 and 21 dpi. Colonized roots by *R*. *tropici* were histochemically analyzed for GUS activity according to Jefferson et al. (1987), and images were acquired with an inverted microscope (Nikon TE300) at 10–40× magnification.

### Expression analysis by RT-qPCR

Total RNA was isolated from leaves using TRIzol reagent (Invitrogen) following the manufacturer’s protocol. To eliminate contaminating genomic DNA, total RNA samples (1 μg in 20 μL) were treated with 1 unit of DNaseI (RNase-free; Invitrogen) at 37°C for 30 min and then at 65°C for 10 min. RT-qPCR was performed using Maxima SYBR Green/ROX qPCR Master Mix (Thermo Fisher, Carlsbad, CA, USA) and quantified on a real-time PCR thermal cycler (QuantStudioTM 5 System, Waltham, MA, USA). Each reaction included *Taq* polymerase, 10 x Taq buffer, and gene-specific primers S3 Table, and 20 ng cDNA in a 10μL final volume. The RT-qPCR was 35 cycles of 95°C for 15 s, 55°C for 30 s, and 72°C for 30 s. Primer specificity was verified by regular PCR and melting curve analysis. *P*. *vulgaris* elongation factor 1- α (PvEf1-α) was used as an internal control for normalization S3 Table. The results were evaluated by the 2−^ΔCT^ method and presented as relative expression based on the cycle threshold difference between each target gene and the *PvEf1-α* in accordance to 2−ΔCT method [72]. RT-qPCR data are averages of three biological replicates with three technical replicates, and two independent experiments were performed.

### ROS determination

Composite *P*. *vulgaris* plants were grown in glass tubes (15 cm) containing Fahraeus nutrient solution to determine O^2-^ levels in transgenic roots at 15 days post emergence. *In situ* O^2-^ was estimated using the nitroblue tetrazolium (NBT) staining method [35]. Samples were incubated for 1 h in darkness at room temperature, the roots cleared in 96% ethanol, and then placed in a 50% glycerol. The presence of O^2-^ was determined with a stereomicroscope (Olympus SZX7, Hamburg, Germany) to observe insoluble blue formazan precipitate. Superoxide was determined by incubating roots with the fluorescent probe 5(6)-carboxy-2′,7-dichlorofluorescein diacetate (H_2_CDFDA (H_2_O_2_) as described [73]. The roots were also placed in a 50% glycerol and the fluorescence observed using a stereomicroscope (Olympus SZX7) using an excitation wavelength of 488 nm and observation wavelength of 525 nm.

### Statistical analysis

Data were processed to obtain the means and standard deviations. The optimal distribution intervals in the histograms were obtained using the Sturge´s rule (Scott, 2011). Significant differences in each parameter were subjected one-way analysis of variance (ANOVA) with *post-hoc* Tukey HSD test. The data used for ANOVA was checked for normal distributions (Shapiro-Wilk’s test) before statistical analysis. The paired Student’s t-test was used to evaluate the significance of differences in the gene expression. Statistically significant differences are represented by the number of asterisks, simple (*) *p*<0.05, double (**) *p*<0.01 and triple (***) *p*<0.001. The statistical analyses were performed using GraphPad Prism v 6 for Windows.

## Supporting information

S1 Fig Surface models for LLG1 and LLG2 proteins of *M*. *truncatula*, *L*. *japonicus*, *C*. *arietinum*, *L*. *culinaris*, *A*. *hypogea* and *G*. *max*. The exposed 13 aa involved in the RALF23 binding are indicated in red, and the exposed amino acids of the conserved motif KEGKEGLE/D is indicated in gray.
(TIF)

S2 Fig *PvLLG1* and *PvLLG2* transcript accumulation in several legumes during nodule development. In panel A, a heatmap is presented with the transcription accumulation values of *PvLLG1* and *PvLLG2* in different tissues and stages of nodule, root, leaf, seed, and pod development. The values are represented in CPM (Counts Per Million). In panel B, a heatmap is presented with the transcript accumulation values of *PvLLG1* and *PvLLG2* in different tissues during the interaction of the plants with the pathogens, *Pseudomonas*, *Edornavirus* and *Fusarium*. In panel C, a heatmap is presented with the transcript accumulation values of LLG genes in *Glycine max* at different stages of nodule development following inoculation with *Bradyrrhizobium diazoefficiens* [42]. In panel D, UMAP (Uniform Manifold Approximation and Projection) diagrams are shown for the tissue-specific expression of LLG genes in *Medicago truncatula* during nodule formation following inoculation with *Ensifer (Sinorhizobium) meliloti* [43].
(TIF)

S3 Fig *PvLLG1* mRNA levels in *P*. *vulgaris* tissues. RNA extracted from denodulated roots or isolated roots. Quantification by RT-qPCR is given relative expression levels (2^−ΔCt^) and was calculated after normalization to the *P*. *vulgaris* housekeeping *PvEf1-α* gene. For each sample, three biological replicates, each were analyzed with two technical replicates. Different letters indicate significant differences among samples according to the ANOVA analysis at *p <0*.*0001*.
(TIF)

S4 Fig Histological examination of nodules of the different constructions used. (**A**) Transmitted light and epifluorescence images of 21-day-old nodules expressing the *pPvLLG*:*GFP-GUS* construct and inoculated with *R*. *tropici-DS RED*, and transmitted-light and epifluorescence images of 21-day-old nodules expressing the *pPvLLG*:*GFP-GUS* construct without *R*. *tropici* inoculation. (**B**) Transmitted light and epifluorescence images of 21-day-old mature nodules expressing the *RNAi*:*PvLLG1* construct and inoculated with *R*. *tropici-GFP*. (**C**) Transmitted light and epifluorescence images of 21-day-old mature nodules expressing the *35S*::*PvLLG1-GFP* construct and inoculated with *R*. *tropici-DS RED*.
(TIF)

S5 Fig Dry weight of root nodules with silencing and overexpression of *PvLLG1*. 21-day-old nodules of roots expressing the *SAC*, *RNAi*:*PvLLG1*, *35S*::*GFP*, and *35S*::*PvLLG-GFP* constructs. The average weight is shown from 3 experiments with all nodules measured on 5 plants per experiment. Letters above the bars represent the significant difference between treatments, determined by an ANOVA with the Fisher test *p<0*.*05*.
(TIF)

S6 Fig ROS distribution in *PvLLG1* silenced and overexpressing *P*. *vulgaris* roots. (**A**) Control (SAC) and (**B**) *PvLLG1* silenced (*RNAi*:*PvLLG1* construct) root with NBT staining to indicate superoxide distribution. (**C**) control (SAC) root labeled with H_2_CDFDA fluorescent probe and (**D**) *PvLLG1* silenced (*RNAi*:*PvLLG1*) indicates the ROS level. (**E**) control root showing the root hair and (**F**) shows the equivalent region under silencing condition. (**G** and **H**) roots with NBT staining under control and overexpression of *PvLLG1*, respectively. (**I** and **J**) roots labeled with H_2_CDFDA fluorescent probe depict the ROS level in control and overexpression condition. (**K** and **L**) root hairs under control and overexpression condition, respectively.
(TIF)

S1 Table E-values and percentage amino acid similarity between the LREs, and LLGs of *A*. *thaliana*, *M*. *truncatula*, *L*. *japonicus*, *C*. *arietinum*, *L*. *culinaris*, *A*. *hypogea*, *G*. *max and P*. *vulgaris*. (PDF)

S2 Table E-values and percentage amino acid similarity between the LREs, and LLGs of *A*. *thaliana*, *M*. *truncatula*, *L*. *japonicus*, *C*. *arietinum*, *L*. *culinaris*, *A*. *hypogea*, *G*. *max and P*. *vulgaris*. (PDF)

S3 Table Primers used for vectors construction and RT-qPCRs. (PDF)
