# Supplementary figures and images for: Role of a LORELEI- like gene from Phaseolus vulgaris during a mutualistic interaction with Rhizobium tropici
Source: PLoS One. 2023 Dec 7;18(12):e0294334. doi: 10.1371/journal.pone.0294334 (PMC10703324; doi:10.1371/journal.pone.0294334)

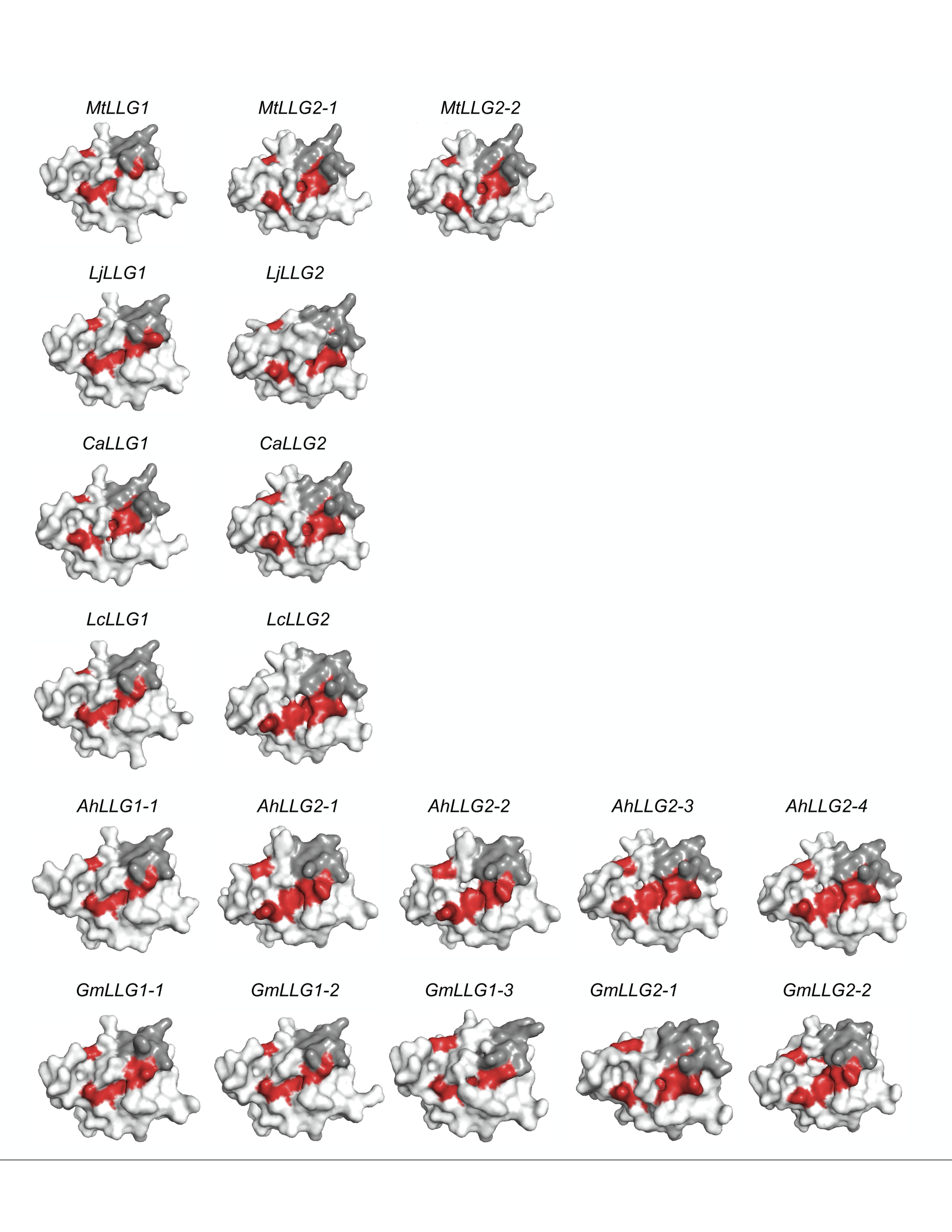

Supplement: S1 Fig — The exposed 13 aa involved in the RALF23 binding are indicated in red, and the exposed amino acids of the conserved motif KEGKEGLE/D is indicated in gray. (TIF) [file pone.0294334.s001.tif]

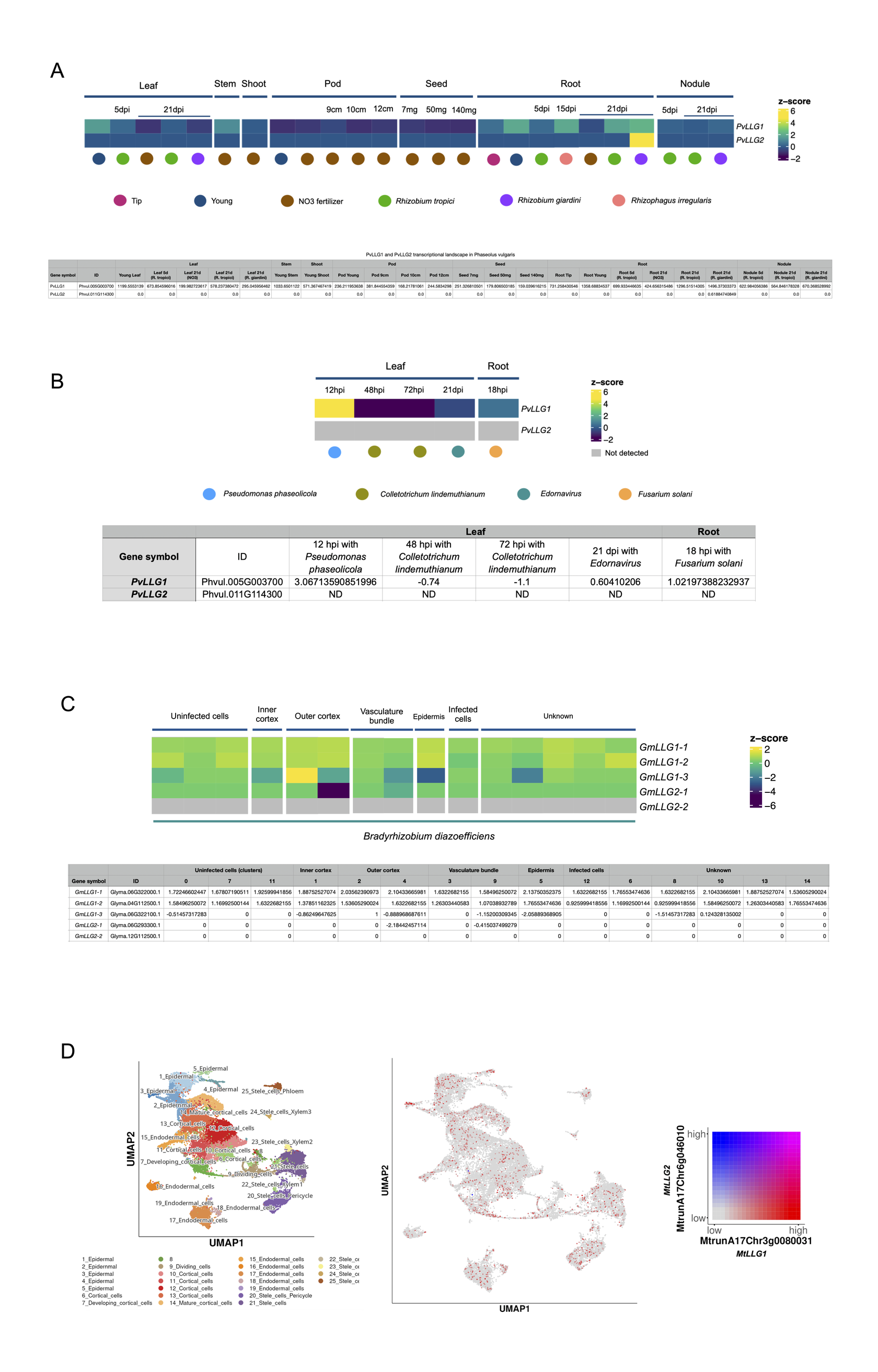

Supplement: S2 Fig — In panel A, a heatmap is presented with the transcription accumulation values of PvLLG1 and PvLLG2 in different tissues and stages of nodule, root, leaf, seed, and pod development. The values are represented in CPM (Counts Per Million). In panel B, a heatmap is presented with the transcript accumulation values of PvLLG1 and PvLLG2 in different tissues during the interaction of the plants with the pathogens, Pseudomonas, Edornavirus and Fusarium. In panel C, a heatmap is presented with the transcript accumulation values of LLG genes in Glycine max at different stages of nodule development following inoculation with Bradyrrhizobium diazoefficiens [42]. In panel D, UMAP (Uniform Manifold Approximation and Projection) diagrams are shown for the tissue-specific expression of LLG genes in Medicago truncatula during nodule formation following inoculation with Ensifer (Sinorhizobium) meliloti [43]. (TIF) [file pone.0294334.s002.tif]

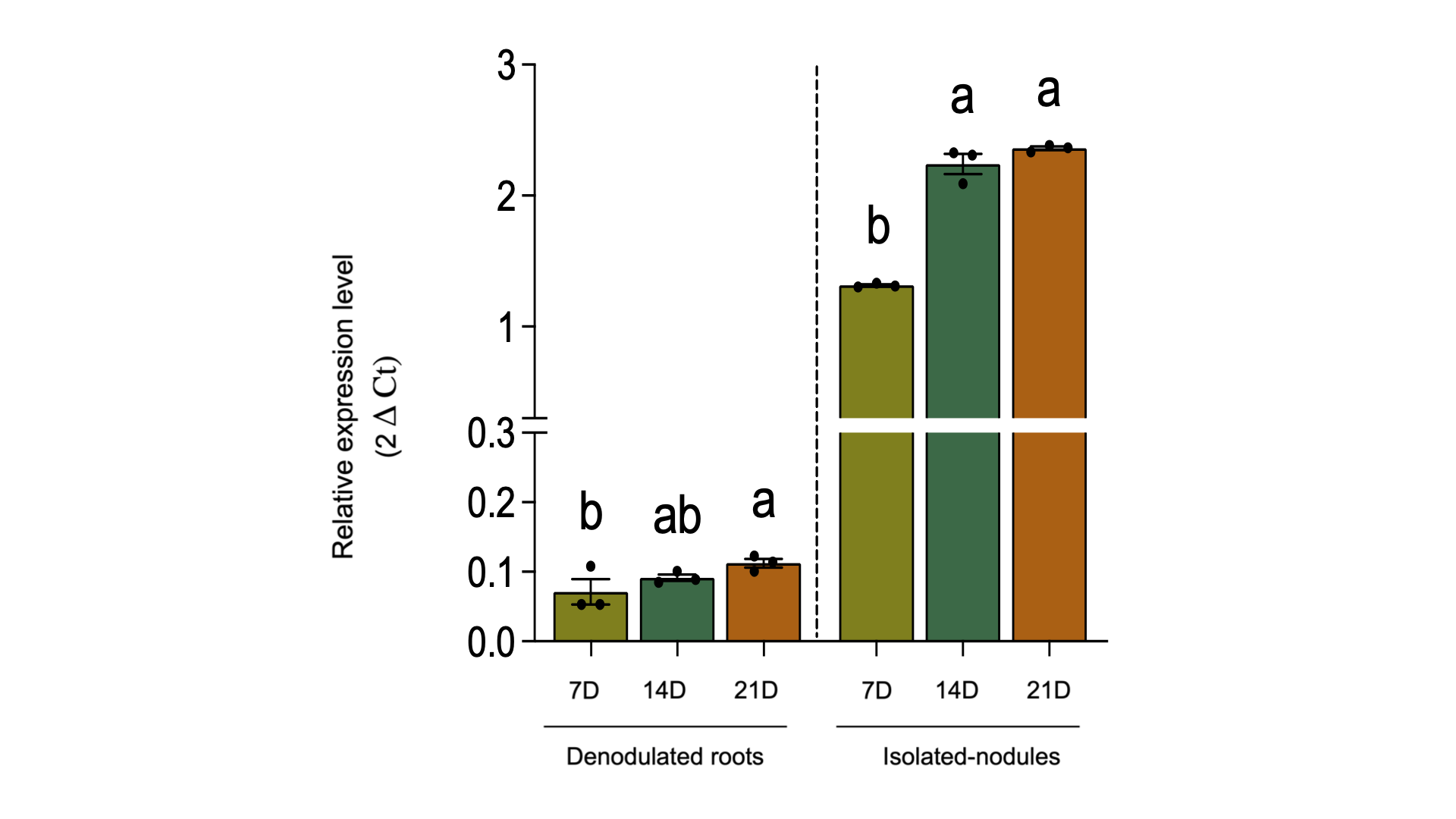

Supplement: S3 Fig — RNA extracted from denodulated roots or isolated roots. Quantification by RT-qPCR is given relative expression levels (2−ΔCt) and was calculated after normalization to the P. vulgaris housekeeping PvEf1-α gene. For each sample, three biological replicates, each were analyzed with two technical replicates. Different letters indicate significant differences among samples according to the ANOVA analysis at p <0.0001. (TIF) [file pone.0294334.s003.tif]

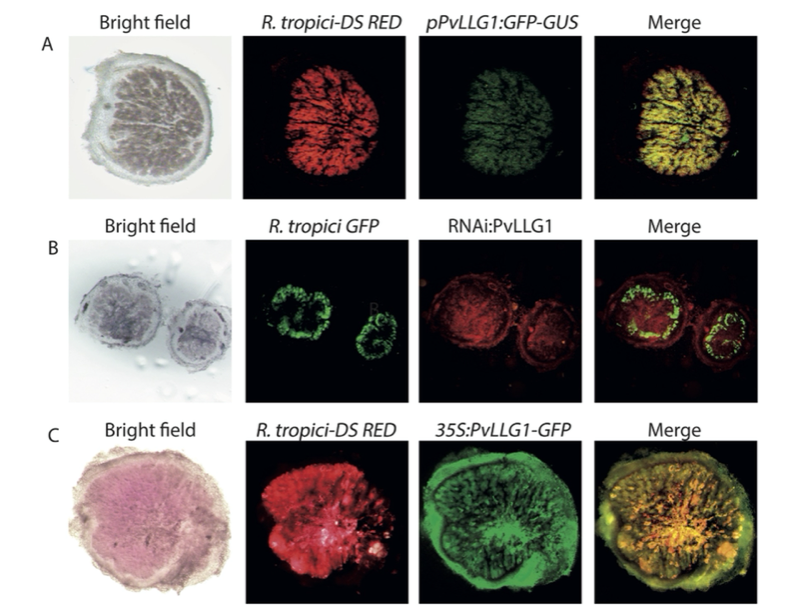

Supplement: S4 Fig — (A) Transmitted light and epifluorescence images of 21-day-old nodules expressing the pPvLLG:GFP-GUS construct and inoculated with R. tropici-DS RED, and transmitted-light and epifluorescence images of 21-day-old nodules expressing the pPvLLG:GFP-GUS construct without R. tropici inoculation. (B) Transmitted light and epifluorescence images of 21-day-old mature nodules expressing the RNAi:PvLLG1 construct and inoculated with R. tropici-GFP. (C) Transmitted light and epifluorescence images of 21-day-old mature nodules expressing the 35S::PvLLG1-GFP construct and inoculated with R. tropici-DS RED. (TIF) [file pone.0294334.s004.tif]

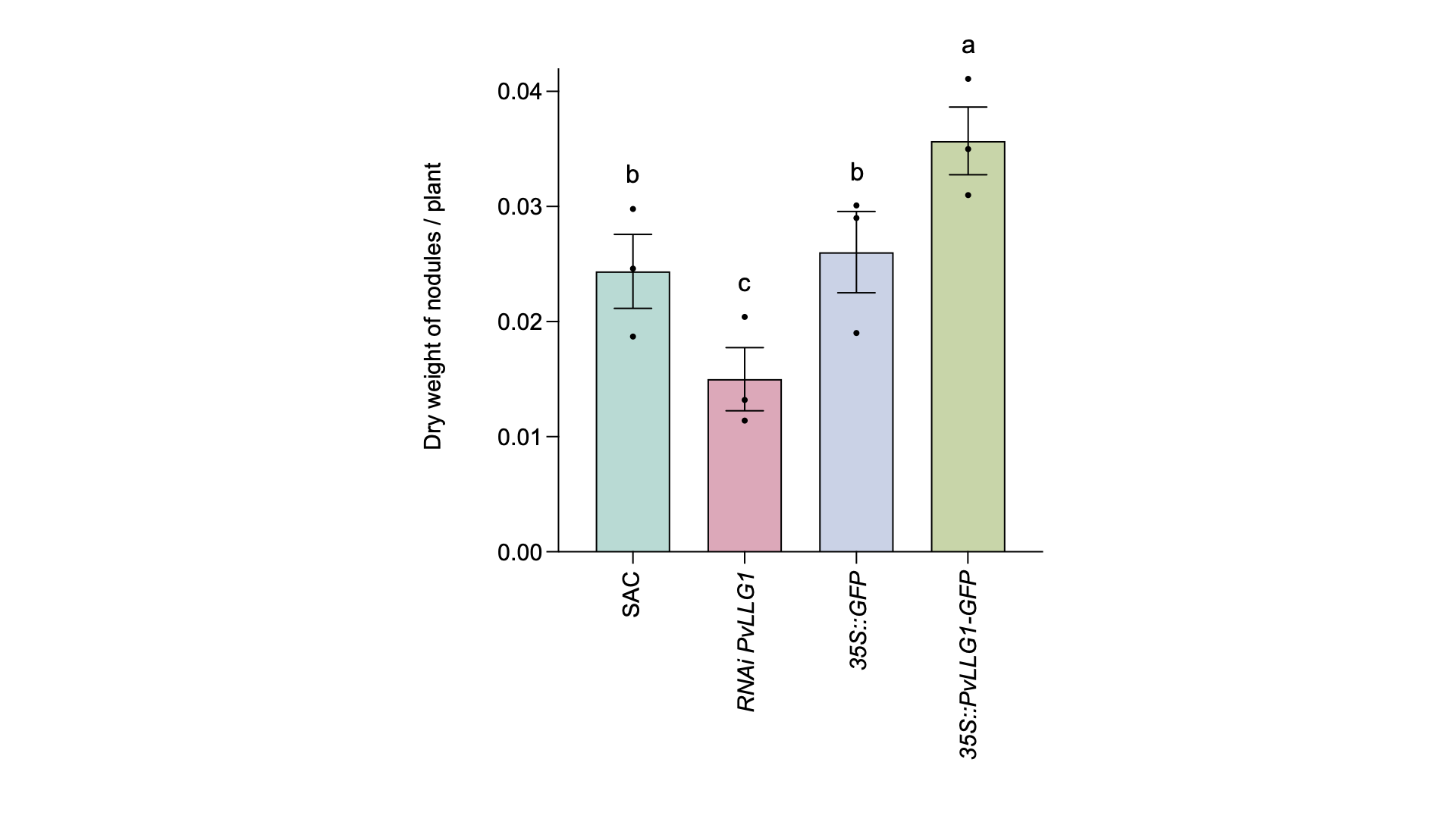

Supplement: S5 Fig — 21-day-old nodules of roots expressing the SAC, RNAi:PvLLG1, 35S::GFP, and 35S::PvLLG-GFP constructs. The average weight is shown from 3 experiments with all nodules measured on 5 plants per experiment. Letters above the bars represent the significant difference between treatments, determined by an ANOVA with the Fisher test p<0.05. (TIF) [file pone.0294334.s005.tif]

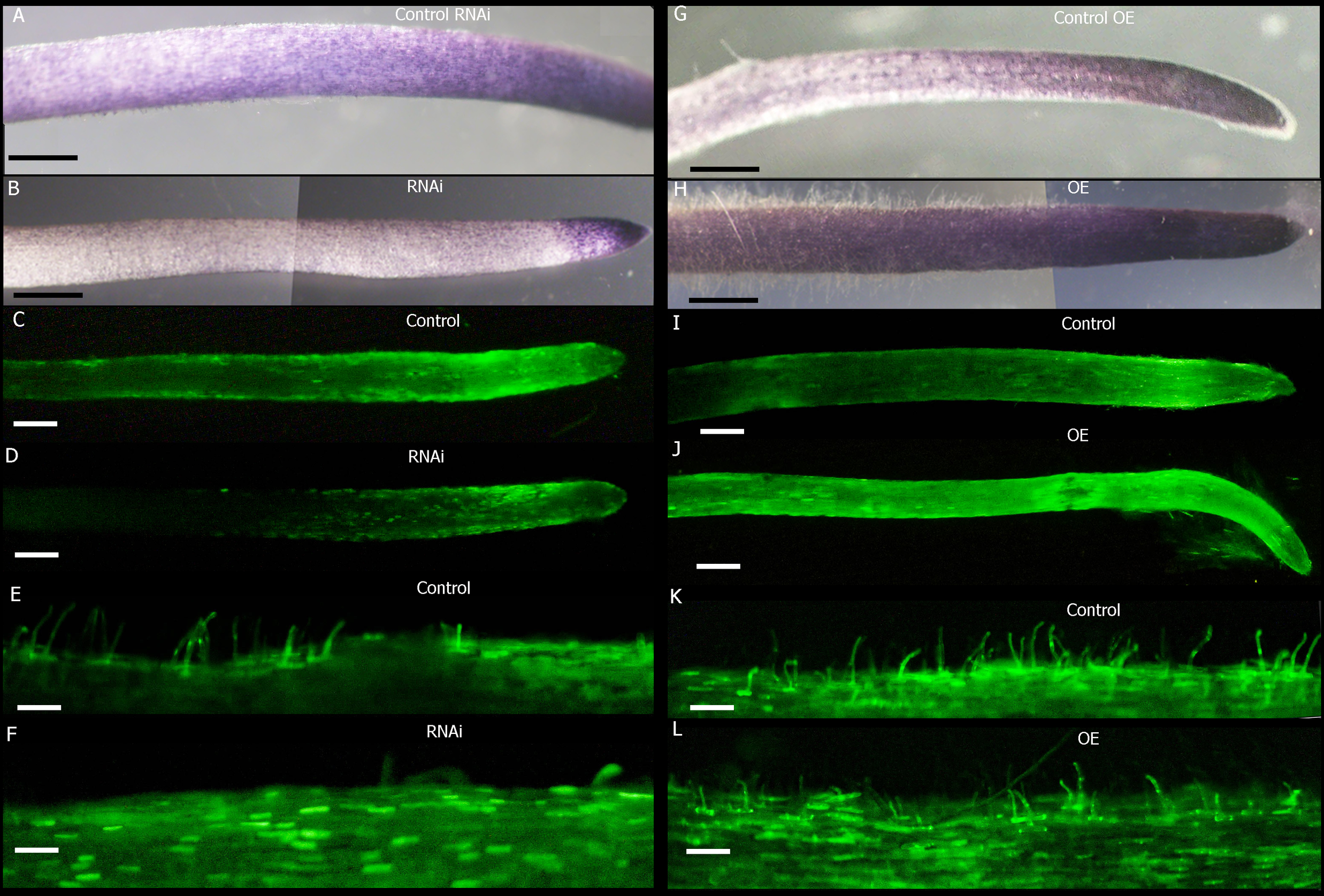

Supplement: S6 Fig — (A) Control (SAC) and (B) PvLLG1 silenced (RNAi:PvLLG1 construct) root with NBT staining to indicate superoxide distribution. (C) control (SAC) root labeled with H2CDFDA fluorescent probe and (D) PvLLG1 silenced (RNAi:PvLLG1) indicates the ROS level. (E) control root showing the root hair and (F) shows the equivalent region under silencing condition. (G and H) roots with NBT staining under control and overexpression of PvLLG1, respectively. (I and J) roots labeled with H2CDFDA fluorescent probe depict the ROS level in control and overexpression condition. (K and L) root hairs under control and overexpression condition, respectively. (TIF) [file pone.0294334.s006.tif]
