# Supplementary material for: Role of a LORELEI- like gene from Phaseolus vulgaris during a mutualistic interaction with Rhizobium tropici
Source: PLoS One. 2023 Dec 7;18(12):e0294334. doi: 10.1371/journal.pone.0294334 (PMC10703324; doi:10.1371/journal.pone.0294334)
Supplement: S1 Table — (PDF) [file pone.0294334.s007.pdf]

E values for PvLLG1 and PvLLG2				
**Gene ID**	**Genome**	**E-value**	**% identity**	**Query ID**
Glyma.04G112500.1.p LLG1-2	G.max Wm82.a4.v1	8.87E-77	85	PvLLG1 | Phvul.005G003700.1.p
Glyma.06G322000.1.p LLG1-1	G.max Wm82.a4.v1	7.89E-75	85	PvLLG1 | Phvul.005G003700.1.p
Medtr3g010180.1 LLG1	M.truncatula Mt4.0v1	1.72E-74	81	PvLLG1 | Phvul.005G003700.1.p
Lj3g0015779.1 LLG1	L.japonicus Lj1.0v1	2.42E-71	84	PvLLG1 | Phvul.005G003700.1.p
Ca_20524 LLG1	C.arietinum v1.0	2.72E-71	79	PvLLG1 | Phvul.005G003700.1.p
arahy.Tifrunner.gnm1.ann1.HTAW4M.5 LLG1	A.hypogaea v1.0	1.07E-69	80	PvLLG1 | Phvul.005G003700.1.p
Lcu.2RBY.3g001120.1 LLG1	L.culinaris v1	4.19E-69	76	PvLLG1 | Phvul.005G003700.1.p
AT5G56170.1 LLG1	A.thaliana Araport11	1.4E-56	77	PvLLG1 | Phvul.005G003700.1.p
Glyma.06G322100.1.p LLG1-3	G.max Wm82.a4.v1	3.98E-56	70	PvLLG1 | Phvul.005G003700.1.p
Ca_22993 LLG2	C.arietinum v1.0	1.91E-53	66	PvLLG1 | Phvul.005G003700.1.p
Glyma.06G293300.1.p LLG2-1	G.max Wm82.a4.v1	3.86E-52	60	PvLLG1 | Phvul.005G003700.1.p
Glyma.12G112500.1.p LLG2-2	G.max Wm82.a4.v1	3.9E-52	60	PvLLG1 | Phvul.005G003700.1.p
Lj3g0014797.1 LLG2	L.japonicus Lj1.0v1	4.36E-52	60	PvLLG1 | Phvul.005G003700.1.p
Lcu.2RBY.2g051320.1 LLG2	L.culinaris v1	3.84E-51	65	PvLLG1 | Phvul.005G003700.1.p
arahy.Tifrunner.gnm1.ann1.GSV8KW.1 LLG2-4	A.hypogaea v1.0	1.9E-47	65	PvLLG1 | Phvul.005G003700.1.p
arahy.Tifrunner.gnm1.ann1.S2IIK5.1 LLG2-3	A.hypogaea v1.0	5.66E-47	64	PvLLG1 | Phvul.005G003700.1.p
Medtr6g018540.1 LLG2-1	M.truncatula Mt4.0v1	4.8E-46	66	PvLLG1 | Phvul.005G003700.1.p
Medtr6g025670.1 LLG2-2	M.truncatula Mt4.0v1	4.8E-46	66	PvLLG1 | Phvul.005G003700.1.p
AT4G26466.1 LRE	A.thaliana Araport11	2.27E-44	62	PvLLG1 | Phvul.005G003700.1.p
AT2G20700.1 LLG2	A.thaliana Araport11	7.24E-44	59	PvLLG1 | Phvul.005G003700.1.p
arahy.Tifrunner.gnm1.ann1.Z92MN7.1 LLG2-2	A.hypogaea v1.0	5.87E-39	64	PvLLG1 | Phvul.005G003700.1.p
arahy.Tifrunner.gnm1.ann1.FW5TAU.2 LLG2-1	A.hypogaea v1.0	7.38E-39	64	PvLLG1 | Phvul.005G003700.1.p
AT4G28280.1 LLG3	A.thaliana Araport11	1.49E-38	62	PvLLG1 | Phvul.005G003700.1.p
Glyma.12G112500.1.p LLG2-2	G.max Wm82.a4.v1	1.03E-84	80	PvLLG2 | Phvul.011G114300.1.p
Lj3g0014797.1 LLG2	L.japonicus Lj1.0v1	1.39E-83	77	PvLLG2 | Phvul.011G114300.1.p
Ca_22993 LLG2	C.arietinum v1.0	1.57E-83	78	PvLLG2 | Phvul.011G114300.1.p
Glyma.06G293300.1.p LLG2-1	G.max Wm82.a4.v1	4.53E-83	79	PvLLG2 | Phvul.011G114300.1.p
Lcu.2RBY.2g051320.1 LLG2	L.culinaris v1	3.58E-81	76	PvLLG2 | Phvul.011G114300.1.p
arahy.Tifrunner.gnm1.ann1.GSV8KW.1 LLG2-4	A.hypogaea v1.0	5.52E-76	79	PvLLG2 | Phvul.011G114300.1.p
arahy.Tifrunner.gnm1.ann1.S2IIK5.1 LLG2-3	A.hypogaea v1.0	2.82E-75	78	PvLLG2 | Phvul.011G114300.1.p
Medtr6g018540.1 LLG2-1	M.truncatula Mt4.0v1	4.14E-65	68	PvLLG2 | Phvul.011G114300.1.p
Medtr6g025670.1 LLG2-2	M.truncatula Mt4.0v1	4.14E-65	68	PvLLG2 | Phvul.011G114300.1.p
Glyma.04G112500.1.p LLG1-2	G.max Wm82.a4.v1	8.24E-48	59	PvLLG2 | Phvul.011G114300.1.p
arahy.Tifrunner.gnm1.ann1.FW5TAU.2 LLG2-1	A.hypogaea v1.0	3.28E-47	60	PvLLG2 | Phvul.011G114300.1.p
arahy.Tifrunner.gnm1.ann1.Z92MN7.1 LLG2-2	A.hypogaea v1.0	3.99E-47	59	PvLLG2 | Phvul.011G114300.1.p
Lj3g0015779.1 LLG1	L.japonicus Lj1.0v1	4.6E-47	59	PvLLG2 | Phvul.011G114300.1.p
Glyma.06G322000.1.p LLG1-1	G.max Wm82.a4.v1	1.26E-46	57	PvLLG2 | Phvul.011G114300.1.p
Medtr3g010180.1 LLG1	M.truncatula Mt4.0v1	1.97E-46	55	PvLLG2 | Phvul.011G114300.1.p
AT2G20700.1 LLG2	A.thaliana Araport11	3.76E-46	58	PvLLG2 | Phvul.011G114300.1.p
arahy.Tifrunner.gnm1.ann1.HTAW4M.5 LLG1	A.hypogaea v1.0	1.2E-45	56	PvLLG2 | Phvul.011G114300.1.p
