# Supplementary material for: Role of a LORELEI- like gene from Phaseolus vulgaris during a mutualistic interaction with Rhizobium tropici
Source: PLoS One. 2023 Dec 7;18(12):e0294334. doi: 10.1371/journal.pone.0294334 (PMC10703324; doi:10.1371/journal.pone.0294334)
Supplement: S2 Table — (PDF) [file pone.0294334.s008.pdf]

AT5G56170.1 LLG1	A.thaliana Araport11	2.2E-45	66	PvLLG2 | Phvul.011G114300.1.p
Ca_20524 LLG1	C.arietinum v1.0	1.74E-44	53	PvLLG2 | Phvul.011G114300.1.p
Lcu.2RBY.3g001120.1 LLG1	L.culinaris v1	7.62E-44	57	PvLLG2 | Phvul.011G114300.1.p
Glyma.06G322100.1.p LLG1-3	G.max Wm82.a4.v1	1.03E-41	63	PvLLG2 | Phvul.011G114300.1.p
AT4G28280.1 LLG3	A.thaliana Araport11	7.73E-40	62	PvLLG2 | Phvul.011G114300.1.p
AT4G26466.1 LRE	A.thaliana Araport11	1.99E-38	58	PvLLG2 | Phvul.011G114300.1.p
