# Supplementary material for: Role of a LORELEI- like gene from Phaseolus vulgaris during a mutualistic interaction with Rhizobium tropici
Source: PLoS One. 2023 Dec 7;18(12):e0294334. doi: 10.1371/journal.pone.0294334 (PMC10703324; doi:10.1371/journal.pone.0294334)
Supplement: S3 Table — (PDF) [file pone.0294334.s009.pdf]

Construction	Oligonucleótide (5´-3´)		Length in base pairs	Amplicon PB	TM (60-65)
qPCR PvLLG1 Phvul.005G003700.1	FW	ATGAGTGTCGTGAAGGGAAGCTT	23	126	60
	RW	AGAAGCAGGTTGTGAGCATGAGC	23		60
qPCR PvLLG2 Phvul.011G114300.1	FW	CTCTATTGTTTCCTCCTAGCCACATTGG	28	135	60
	RW	CTGGTTCTCAAAGTCAACTGCACA	24		59
2Kb pPvLLG1	FW	caccGTATGAGATCCATGACTTTATATTGAGGAGGC	54	2000	60
	RW	ACACAACAAAACTGAGGAACAACATAAATAT-TATTTTTTGTT	42		60
35S PvLLG1	FW	caccATGGTGTTTTCTGCCCACCAACG	27	507	61
	RW	GAATAACAAAATTAAGAAGCAGGTTGTGAGC	31		50
RNAi PvLLG1	FW	caccGGAAAAAAGACAGCTCATAAGAACATGTTTGC	36	224	65
	RW	AGTAATGTATCTGATTTGAGTAACGGGTAAAGAGAA	36		61
PvEF1α	FW	GGTCATTGGTCATGTCGACTCTGG	24	146	60
	RW	GCACCCAGGCATACTTGAATGACC	24		60
